# Inhibitory effect of a weight-loss Chinese herbal formula RCM-107 on pancreatic α-amylase activity: Enzymatic and *in silico* approaches

**DOI:** 10.1371/journal.pone.0231815

**Published:** 2020-04-29

**Authors:** Shiqi Luo, George Binh Lenon, Harsharn Gill, Andrew Hung, Daniel A. Dias, Mingdi Li, Linh Toan Nguyen

**Affiliations:** 1 School of Health and Biomedical Sciences, RMIT University, Victoria, Australia; 2 School of Science, RMIT University, Victoria, Australia; 3 Department of Endocrine, Vietnam Military Medical University, Hanoi, Vietnam; University of Akron, UNITED STATES

## Abstract

Reducing carbohydrates digestion by having a low glycaemic index (GI) foods has been linked to weight loss. Inhibiting related enzymes is an alternative way to decrease carbohydrate digestion. RCM-107 (Slimming Plus), an eight-herb formula that is modified from RCM-104, indicated significant weight-loss action in clinical trials. However, no published research has studied its mechanism of action on reducing carbohydrate absorption via suppressing the activities of porcine pancreatic alpha-amylase (PPA). In this paper, we used fluorescence PPA inhibition assay to investigate the inhibitory effects of RCM-107 and the individual herbs present in this herbal mixture on amylase activity. Subsequently, molecular docking predicted the key active compounds that may be responsible for the enzyme inhibition. According to our results, both the RCM-107 formula and several individual herbs displayed α-amylase inhibitory effects. Also, marginal synergistic effects of RCM-107 were detected. In addition, alisol B, (-)-epigallocatechin-3-gallate (EGCG) and plantagoside have been predicted as the key active compounds that may be responsible for the α-amylase inhibition effect of RCM-107 according to inter-residue contact analysis. Finally, Glu233, Gln63, His305, Asp300 and Tyr151 are predicted to be markers of important areas with which potential amylase inhibitors would interact. Therefore, our data has provided new knowledge on the mechanisms of action of the RCM-107 formula and its individual herbal ingredients for weight loss, in terms of decreasing carbohydrate digestion via the inhibition of pancreatic alpha-amylase.

## Introduction

Obesity has been defined as a chronic disease by the Obesity Society (TOS) in 2018 due to its emerging epidemiological trend [1]. It increases the risk of developing other metabolic disorders such as hypertension, type 2 diabetes, cardiovascular diseases and myocardial infarction [2, 3]. At least 2.8 million adults die due to being overweight or obese each year [4]. Globally, over 1.9 billion adults were overweight while more than 650 million adults were obese in 2016 [4].

Currently, there is a variety of therapeutic targets available for weight reduction, such as pancreatic lipase, alpha-amylase, glucagon like peptide-1 (GLP-1) receptor and serotonin 2C receptor [5–7]. Along with the general reduction in use of appetite suppressants which act on the central nervous system (CNS), e.g. fenfluramine, d-fenfluramine and rimonabant [5], drugs that act on the periphery have gained wider use [5]. Some periphery-acting drugs have proven successful in weight management with mild intolerances, especially those which reduce the digestion and absorption of nutrients [6].

In humans, various forms of carbohydrates account for between 40% to 80% of total caloric intake [5, 6]. Low GI foods (GI value < 55), the polymeric form of carbohydrates that are absorbed slowly, have been linked to glycemic control and weight loss [8]. An alternative to low GI foods are products that can decrease carbohydrate absorption via suppressing related enzymes such as pancreatic α-amylase [6, 8]. Αlpha-amylase is responsible for catalysing the hydrolysis of α-(1, 4)-glycosidic linkages of starch components and glycogen. Therefore, suppressing this enzyme could result in a general decrease of the main dietary carbohydrates absorption [5]. The known alpha-amylase inhibitors such as acarbose have been used as an off-label agent to assist weight loss [9]. In addition, the supplement Phase2^®^ *Phaseolus vulgaris* white bean extract demonstrated weight-loss effects in human clinical trials via its amylase inhibitory activity [10].

Chinese herbal formulas are therapeutic herbs traditionally used in combination rather than individually. Many *in vitro* and *in vivo* studies [11–16] have demonstrated the synergistic actions of herbal formulas, indicating that herbal formulas show significantly better pharmacological effects than single herbs for different conditions, including obesity [17]. Park et al. [15] reported a stronger weight-reducing effect of two herbs (*Panax ginseng* and *Veratrum nigrum)* used in combination rather than separately on high-fat diet induced mice. Active classes such as phenolic and flavonoid contents from a given herbal medicine can be responsible for their synergistic effects [17]. However, it is challenging to evaluate these synergistic effects accurately as the exact chemical, and pharmacological properties of Chinese herbs are not clearly defined [18]. An integrated systematic analysis approach, including literature, experimental and computational studies have been developed to assist analyzing complex multi-ligands-targets synergistic actions [17, 18].

Additionally, molecular docking has been commonly used in drug design, aiming to predict binding sites of a ligand with a target protein. The effect of a ligand on the target protein could be predicted by comparing its binding site with an established drug (e.g. inhibitor), which has known action on that protein. Also, molecular docking has been applied to identify key active compounds that can bind to the corresponding targets at the known active site [18].

In this paper, we have investigated the RCM-107 formula (Slimming Plus), modified from our previous studied RCM-104 formula, which demonstrated significant effects on weight loss in clinical trials [19]. RCM-107 contains eight Chinese herbs, including *Camellia sinensis /* Lu cha ye; *Poria* / *Poria cocos (schw*.*) Wolf*. / Fu ling; Nelumbinis *folium* / *Nelumbo nucifera Gaertn*. */* He ye*; Alismatis rhizome / Alisma orientalis (sam*.*) Juzep*. */* Ze xie; *Plantaginis semen* / *Plantago asiatica L*. */* Che qian zi; Cassiae semen / *Cassia obtusifolia L*. */* Jue ming zi; *Sophorae flos* / *Sophora japonica L*. */* Huai hua and *Gardeniae fructus* / *Gardenia jasminoides Ellis*. / Zhi zi [20]. Our recent work has demonstrated potent pancreatic lipase inhibitory effect of this formula, which may lead to a decrease of lipid absorption, thus weight loss. Also, we have identified the presence of six active chemical compounds in RCM-107 and related single herbs, such as EGCG, epicatechin-3-gallate (ECG), (-)-epicatechin (EC), caffiene, rutin and crocin [21]. However, to date there is no published scientific evidence indicating the effects of the RCM-107 and its individual herbal ingredients on pancreatic alpha-amylase activity.

Therefore, this present study aims to firstly determine the effects of RCM-107 and individual herbs in this formula on the inhibition of porcine pancreatic alpha-amylase (PPA) using experimental amylase inhibition assays. The porcine form of the enzyme is an appropriate choice for this work due to the high sequence similarity between human, porcine and mouse pancreatic alpha-amylase [22]. The results from the present study can therefore readily be extrapolated to understand the mechanisms of action of the formula on a range of mammals, including humans.

In addition, key active components that could act as potential PPA inhibitors from the individual compounds within these herbs in the RCM-107, obtained from the literature [20, 23–35], will be predicted via molecular docking analyses.

## 2. Materials and methods

### 2.1 Amylase inhibition assay

#### 2.1.1 Materials

A EnzCheck^®^ Ultra Amylase Assay Kit (E33651) and phosphate buffer saline (PBS) tablets (18912) were obtained from Life Technologies Australia Pty Ltd (Mulgrave, AUS). Porcine pancreatic α-amylase (PPA) (50 mg/5mL, 10102814001), Dimethylsulfoxide (DMSO, 472301-500mL) and acarbose (A8980-1G) were purchased from Sigma-Aldrich (Australia). RCM-107 capsules (15733, AUST L 285569) were provided by Tong Kang Lee Chinese Medical Centre (Kensington VIC Australia) and herbal granules (Nong’s, HK) consisting of *Camellia sinensis /* matcha */* Lu cha ye; *Poria* / *Poria cocos (schw*.*) Wolf*. / Fu ling (A1601642; 1006A1601642031019); *Nelumbinis folium* / *Nelumbo nucifera Gaertn*. */* He ye (A1601450; 1356A1601450060919)*; Alismatis rhizome / Alisma orientalis (sam*.*) Juzep*. */* Ze xie (A1600004; 1015A1600004070119)*; Plantaginis semen* / *Plantago asiatica L*. */* Che qian zi (A1600813; 1072A1600813080819); Cassiae semen / *Cassia obtusifolia L*. */ Jue ming zi* (A1500060; 1161A1500060040218); *Sophorae flos* / *Sophora japonica L*. */* Huai hua (A1601428; 1203A1601428071119) and *Gardeniae fructus* / *Gardenia jasminoides Ellis*. / Zhi zi (A1600810; 1033A1600810050719) were supplied by GL natural health care clinic (Strathmore, VIC, Australia).

#### 2.1.2. Sample preparation

Each RCM-107 capsule contains 500 mg extracted herbal powder (including 150mg of *Camellia sinensis* and 50mg of the rest seven ingredients) mixed with 180mg excipients, such as silica colloidal anhydrous, calcium hydrogen phosphate dihydrate, magnesium stearate and microcrystalline cellulose. Herbal powder and granules were obtained by water extraction of the raw materials via boiling and lyophilisation. The ratio between extracted: crude/dry herbs is 1:10. Twenty milligrams of the RCM-107 herbal powder and eight herbal granules were weighed and dissolved in 4 mL of distilled water contained 2% DMSO. The sample was vortexed for 5 min and sonicated for 10 min then filtered through a Millex-HP 0.45μm filter (Millipore). Dilution from the stock solution (5 mg/mL) was performed to achieve a final concentration of 300 μg/mL. In addition, a serial dilution of the RCM-107 formula was conducted to yield a seven calibration point ranging from 10–800μg/mL. The positive control acarbose was prepared in the same manner.

#### 2.1.3 Fluorescence assay

Fluormetric assay of PPA activity using DQ^™^ Starch as a substrate was determined using a modified method previously described by Yilmazer et al. [36]. Twenty-five microliters of each individual herbal extract at the final concentration of 300 μg/mL, the RCM-107 formula extraction (10–800μg/mL) and 25 μL of the PPA solution (final concentration 12mU/mL) were pre-incubated in a 96-well plate with 25 μL PBS buffer (The incubation buffer consisted of 10mM sodium phosphates, 2.68mM KCl, 140 mM NaCl and 1mM CaCl_2_, pH 6.9), in dark at room temperature (25 °C) for 20 min. To initiate the enzyme reaction, 25μL of 200 μg/mL DQ^™^ starch solution was added. Fluorescence was measured with the *CLARIOstar*^®^ microplate reader (BMG labtech) at an excitation and emission wavelength of 485nm and 530nm, respectively over 30 min. Acarbose (a known pancreatic alpha-amylase inhibitor) was used as a positive control. The final concentration of DMSO was 0.16% and showed no effects on PPA activity. The inhibition activity (%) of the PPA was calculated by the following equation:

$$(1-\frac{F\;sample-Fsample\;background-F\;blank}{F\;control-Fcontrol\;background-F\;blank})*100$$

F_sample_ and F_sample_ background represent a fluorescence value of the sample solution with or without substrate, while F_control_ and F_control_ background are a fluorescence value of the control (contains no inhibitors) with or without substrate, respectively. F_blank_ is the fluorescence value of the selected blank, which consists of the substrate and the buffer.

#### 2.1.4. Statistical analysis

Samples and controls were run in triplicates. Results are presented as mean ± standard error from 3 independent experiments. Wells that contain either samples and buffer or enzyme and buffer only were used to detect background auto-fluorescence. The concentration providing 50% inhibition (IC_50_) was calculated by non-linear regression (values are mean ± standard deviation SD) and statistical significance was assessed with one-way analysis of variance (ANOVA) followed by the Tukey multiple comparison test via Graphpad Prism Software version 7. Results with a *P* value < 0.05 were considered statistically significant.

### 2.2 Molecular docking

#### 2.2.1 Compound screening

A total of 149 ligands were obtained from the literature, including the major bioactive chemical constituents from each herb present in RCM-107 [20, 23–35]. Other compounds which met the common requirements for potential favorable therapeutic efficacy, such as good oral bioavailability (OB) ≥ 30%, drug likeness (DL) ≥ 0.18 and intestinal epithelial permeability (Caco-2) ≥ -0.40 were also included [14, 37].

#### 2.2.2 Software and input files

The docking software PyRx version 0.8, together with Autodock Vina, was used for all docking calculations (https://pyrx.sourceforge.io/). The structures of small molecules (herbal ligands, the positive control acarbose and the substrate starch) and macromolecules (the target protein) required to initiate structure-based virtual screening were obtained and pre-processed as follows.

The structures of small molecules were obtained from **Pubchem or TCMSP.** The names of ligands were searched from Pubchem, and their 3D structures were downloaded in the form of SDF files; Subsequently, the Online SMILES Translator and structure file generator (https://cactus.nci.nih.gov/translate/) was then used to translate the SDF files into PDB files, which are recognized by PyRx and Autodock Vina. The canonical SMILES sequences were compared when more than one structure appeared for the same ligand. If the SMILES sequences were different, all structures of this ligand would be collected for docking, otherwise the first structure on the list would be selected.

The PPA structure (PDB CODE: 1OSE) was obtained from the **RCSB Protein Databank** (www.rcsb.org). The 3D protein structures were modified using Visual Molecular Dynamics (VMD) by removing water molecules to obtain protein-only structures, and the prepared structure was saved in PDB format.

#### 2.2.3. Autodock vina

Both selected protein (1OSE) and ligand files were loaded to PyRx as macromolecules and ligand, respectively. Proteins are fixed, while ligands were set to have rotatable torsions. Protein and ligand hydrogens were automatically added using the PyRx hydrogen (H) repair functionality. A box of size X: 8.3130; Y: 73.5614; Z: 146.1870 were defined around the centre of the protein, with the exhaustiveness parameter set to 64 for all dockings. Autodock Vina automatically samples different conformation of the ligands to best fit the predicted binding site. The top 3 highest binding affinity ligands from each herb have been selected for inter-residue interaction analysis. The two-dimensional (2D) ligand and target interaction diagram can be generated by Discovery Studio Visualizer (DSV) Version 4.5, which can be found from BIOVIA (https://www.3dsbiovia.com/products/collaborative-science/biovia-discovery-studio/). The three-dimensional (3D) image and binding sites were observed and displayed in VMD.

## 3. Results and discussion

### 3.1. Amylase inhibition assay

**Fig 1:**
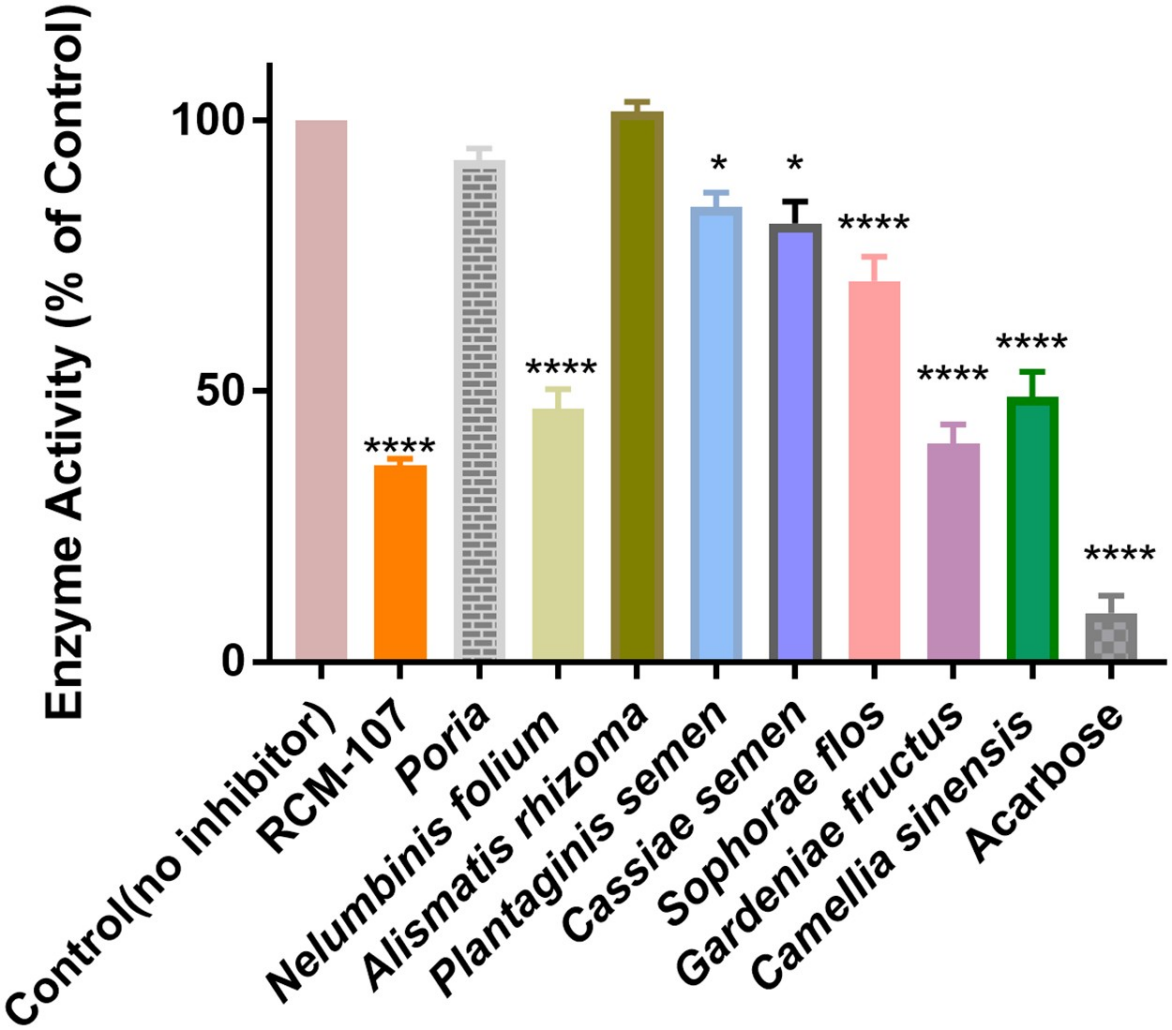
Suppressive effects of the RCM-107 formula, eight single herbal granules and acarbose (inhibitor) at 300 μg mL^-1^ on porcine pancreatic α-amylase activity. The enzyme activity with an absence of the samples or inhibitor was presented as 100%. Data are expressed as means ± standard error of the mean (SEM) from three independent experiments, including three replicates each time. **** indicates *P* < 0.0001 as compared to the control.

The RCM-107 formula and six individual herbs present in this formula displayed significant PPA inhibitory activities after screening at 300μg/mL (Fig 1). The RCM-107 formula exhibited the highest inhibition rate (64%) followed by *Gardeniae fructus*, *Nelumbinis folium* and *Camellia sinensis* (60%, 54% and 51%, respectively). However, our data has shown that differences between those three herbs and the RCM-107 formula were not statistically significant (*P* > 0.05). *Sophorae flos* did present significant PPA inhibition (*P* < 0.0001) but at a much lower inhibition rate (29%) compared to the above four samples. In addition, *Cassiae semen* and *Plantaginis semen* displayed an inhibition rate of 17% and 16%, respectively and were statistically significant (*P < 0*.*05)*.

Only two single herbs *Poria* and *Alismatis rhizome* were statistically *non*-significant (*P* > 0.05) on PPA. *Alismatis rhizome* slightly increased the enzyme activity rather than suppressing it. Even though the inhibitory potency of the RCM-107 formula, *Gardeniae fructus*, *Nelumbinis folium* and *Camellia sinensis* are less active than acarbose, the data suggests they may act as a milder inhibitor of PPA. On the other hand, *Poria* and *Alismatis rhizome* had little or no effects on suppressing this enzyme.

**Fig 2:**
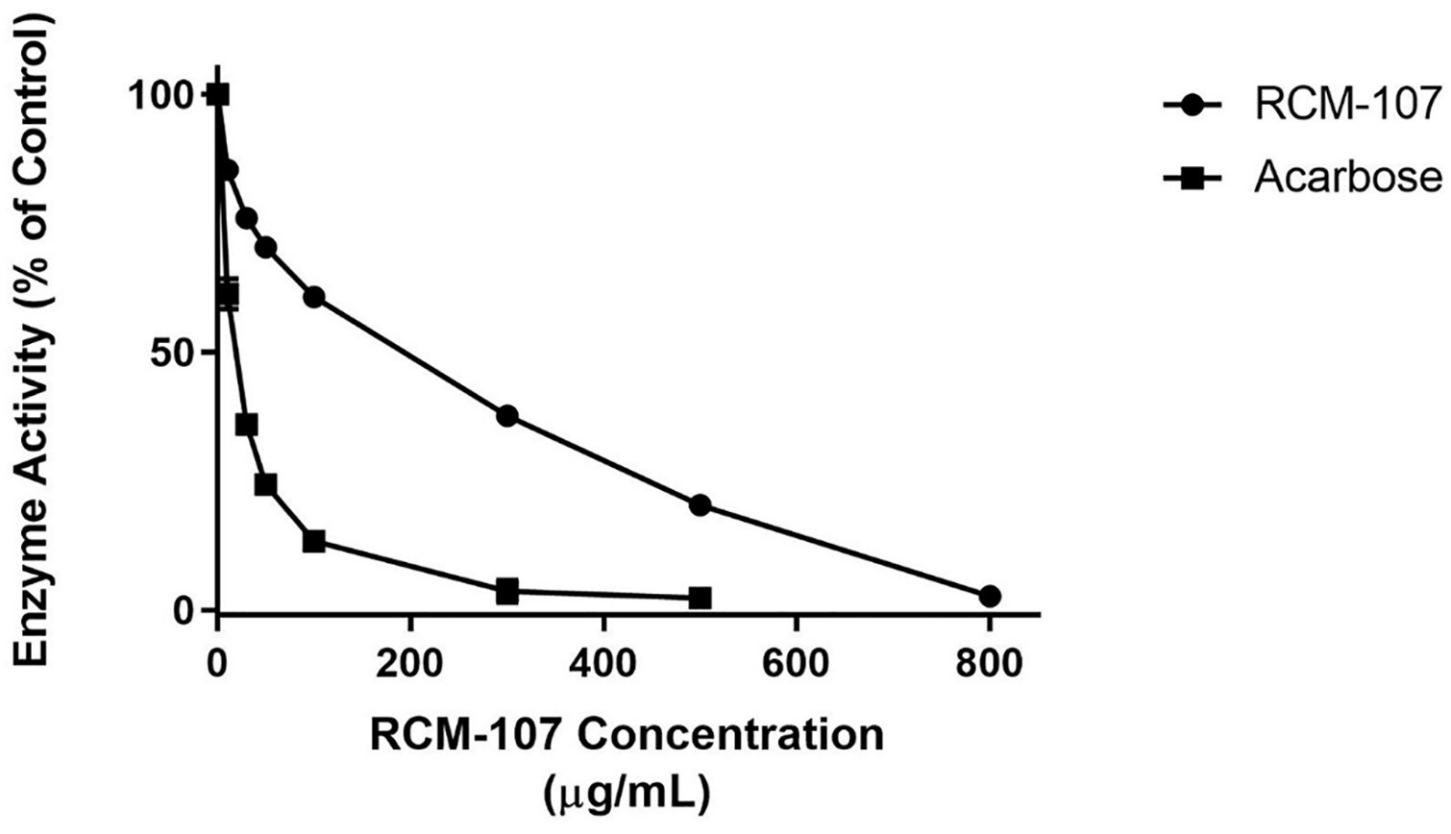
Dose-dependent inhibitory effects of the RCM-107 formula (1–800μg mL^-1^) on porcine α-amylase. Data represent mean ± SEM from three independent experiments with three replicates per condition.

Both the RCM-107 formula and the known amylase inhibitor, acarbose, demonstrated their effects by suppressing PPA in a dose-dependent manner (Fig 2). The enzyme activity was reduced dramatically from 85% to 3% when the concentration of the RCM-107 formula was raised from 10–800 μg/mL. The inhibitory potency of acarbose was presented by achieving the maximum inhibition (98%) at the concentration of 500 μg/mL. Acarbose showed a lower IC_50_ (16.31± 1.74 μg/mL) compared to the RCM-107 formula (119.5 ± 17.14 μg/mL). This data indicates that the RCM-107 formula can inhibit pancreatic α-amylase but not as potently as the positive control.

It is worthy to note that RCM-107 is an 8-herb combination at 3:1(w/w) ratio, in which the proportion of *Camellia sinensis* is 3-fold higher than the other 7 herbs in this formula (a constant value), due to its role as a key herb (“Jun”) [18]. However, in the present amylase inhibition assay, equal concentrations of the herbal formula and individual herbs were used. This difference in the relative proportion of *Camellia sinensis* in the actual formula compared to that in the present assay study may partly contribute to the present lack of observable synergistic effects of RCM-107 in inhibiting PPA enzymatic activity. Nonetheless, it may be proposed that *Gardeniae fructus*, *Nelumbinis folium* and *Camellia sinensis* are the leading herbs in RCM-107 that contribute to the action of α-amylase suppression as they present statistically significant inhibitory effects. In addition, it is proposed that increasing the proportion of individual herbs, such as *Gardeniae fructus* or *Nelumbinis folium*, in the multiple-herbal combination may serve to improve the significance of synergistic effects of RCM-107 [18].

### 3.2 Molecular docking studies and herbal ligand-target interactions

In order to predict the likely main active compounds within the RCM-107 formula which serve to most effectively inhibit alpha-amylase, molecular docking was used to predict the predominant binding mode of potential active components with the selected protein target alpha-amylase [18]. Furthermore, the predicted interactions between bioactive herbal ligands and the targeted protein were compared with a positive control (a known inhibitor, acarbose) as well as an endogenous substrate (starch), in order to propose the key residues that may be required for a potential inhibitor to interact with, and which may therefore be responsible for the suppression of the activity of the protein target [18]. Thus, we take two approaches to propose potentially effective PPA inhibitors from RCM-107: first, by determining ligands which show the highest predicted binding affinity values; and second, by determining those ligands which share the most common interactions with PPA as the positive control, acarbose.

Acarbose was docked to the crystal structure alpha-amylase as a positive control, and three binding poses were predicted to lie at the known active site, which consists of a V-shaped depression located at the carboxyl end of the ‘domain A’ β-barrel [22, 38]. There are four main classes of interactions present in this known α-amylase antagonist (Table 1). Firstly, H-bonds are formed with acidic residues such as Glu233 and Asp300. Secondly, H-bonding with basic residues Lys200 and His305 are also present. Thirdly, interaction with the backbone of Gly residues, such as Gly306. Finally, aromatic ring-ring interactions with Trp58 and Trp59 provides further support to firmly anchor the inhibitor into the active site.

**Table 1:** Top 3 ligands predicted from each herb with the strongest binding affinity and their bonds with IOSE.

Herbal names	Ligands	PubChem ID	Molecular formula	Molecular Weight (g/mol)	Hydrogen bond (amino acids)	Unfavourable bond (amino acids)	Lowest binding affinity (kal/mol)
***Cassia semen***	Stigmasterol (compound **1**)	5280794	C29H48O	412.702	**GLU233**	-	-9.8
***Cassia semen***	Campesterol (compound **2**)	173183	C28H48O	400.691	-	-	-9.5
***Cassia semen***	Cassiaside (compound **3**)	164146	C20H20O9	404.371	**GLN63**	GLN63	-9.2
					GLY106		
					GLY164		
					LEU165(Carbon hydrogen bond)		
					**VAL163** (Carbon hydrogen bond)		
***Camellia sinensis***	Theaflavin (compound **4**)	114777	C29H24O12	564.499	HIS201	**GLN63**	-10
					ASP356		
					**HIS305**		
					**GLY306**		
					TRP59		
***Camellia sinensis***	Thearubigin (compound **5**)	100945367	C43H34O22	902.723	**GLU233** (2 BONDS)	-	-9.8
					**ASP300** (2 BONDS)		
					**HIS305** (2 BONDS)		
					**GLN63**		
					TRP59		
					VAL354		
***Camellia sinensis***	EGCG (compound **6**)	65064	C22H18O11	458.375	**HIS305**	-	-9.5
					**TYR151** (2 BONDS)		
					HIS201		
					**GLU233**		
					ASP197		
					**GLN63**		
***Nelumbinis folium***	Cycloartenol (compound **7**)	92110	C30H50O	426.729	-	-	-9.6
***Nelumbinis folium***	Quercetin (compound **8**)	5280343	C15H10O7	302.238	**ASP300**	-	-9.4
					ASP197		
					TYR62		
					**GLN63**		
					**HIS305**		
***Nelumbinis folium***	Isorhamnetin (compound **9**)	5281654	C16H12O7	316.265	**GLN63** (2 BONDS)	-	-9.2
					TYR62		
					ASP197		
***Sophorae flos***	Beta-sitosterol (compound **10**)	222284	C29H50O	414.718	-	-	-9.6
***Sophorae flos***	N-[6-(acridin-9-ylamino)hexyl]benzamide (compound **11**)	146515	C26H27N3O	397.522	**GLU233** (Carbon hydrogen bond)	-	-9.5
***Sophorae flos***	Sophoradiol (compound **12**)	9846221	C30H50O2	442.728	-	-	-9.4
***Gardeniae fructus***	Sudan III (compound **13**)	62331	C22H16N4O	352.397	**HIS305**	-	-10
***Gardeniae fructus***	Stigmasterol	See above	See above	See above	See above		-9.8
***Gardeniae fructus***	Quercetin	See above	See above	See above	See above		-9.4
***Alismatis rhizome***	Alisol B (compound **14**)	189051	C30H48O4	472.71	**VAL163**	**HIS305**	-10.7
					**GLN63**		
***Alismatis rhizome***	Alisol A (compound **15**)	15558616	C30H50O5	490.725	-	**VAL163**	-10.1
***Alismatis rhizome***	Alisol C (compound **16**)	101306923	C30H46O5	486.693	**GLN63**	**VAL163**	-10
					**HIS305**		
***Poria***	Stellasterol (compound **17**)	5283628	C28H46O	398.675	-	-	-9.7
***Poria***	Eburicoic acid (compound **18**)	73402	C31H50O3	470.738	HIS299	-	-9.6
***Poria***	Dehydroeburicoic acid (compound **19**)	15250826	C31H48O3	468.722	TYR62 (Pi-Doner Hydrogen bond)	-	-9.6
***Plantaginis semen***	Plantagoside (compound **20**)	174157	C21H22O12	466.395	**GLN63**	GLN63	-10.5
					HIS299		
					**GLU233** (2 BONDS)		
					**ASP300**		
					**TYR151**		
					HIS201		
					HIS201(Carbon hydrogen bond)		
					**HIS305**		
***Plantaginis semen***	Daucosterol_qt (compound **21**)	-	-	414.79	-	-	-9.8
***Plantaginis semen***	Quercetin	See above	See above	See above	See above	-	-9.4
**The known amylase inhibitor**	Acarbose (**Reference**)	41774	C25H43NO18	645.608	GLN63	GLN63	-8.4
					GLY104	HIS305	
					ASP300		
					GLU233		
					GLY306		
					GLU240		
					LYS200		
					ASP300		
					VAL163		
					LYS200		
					TYR151		
**Substrate**	Corn starch	24836024	C27H48O20	692.661	HIS305	-	-6.4
					GLY304		
					GLY306		
					TYR151		
					LYS200		
					GLU233		

Their common residues shared with acarbose have been marked in bold.

**Fig 3:**
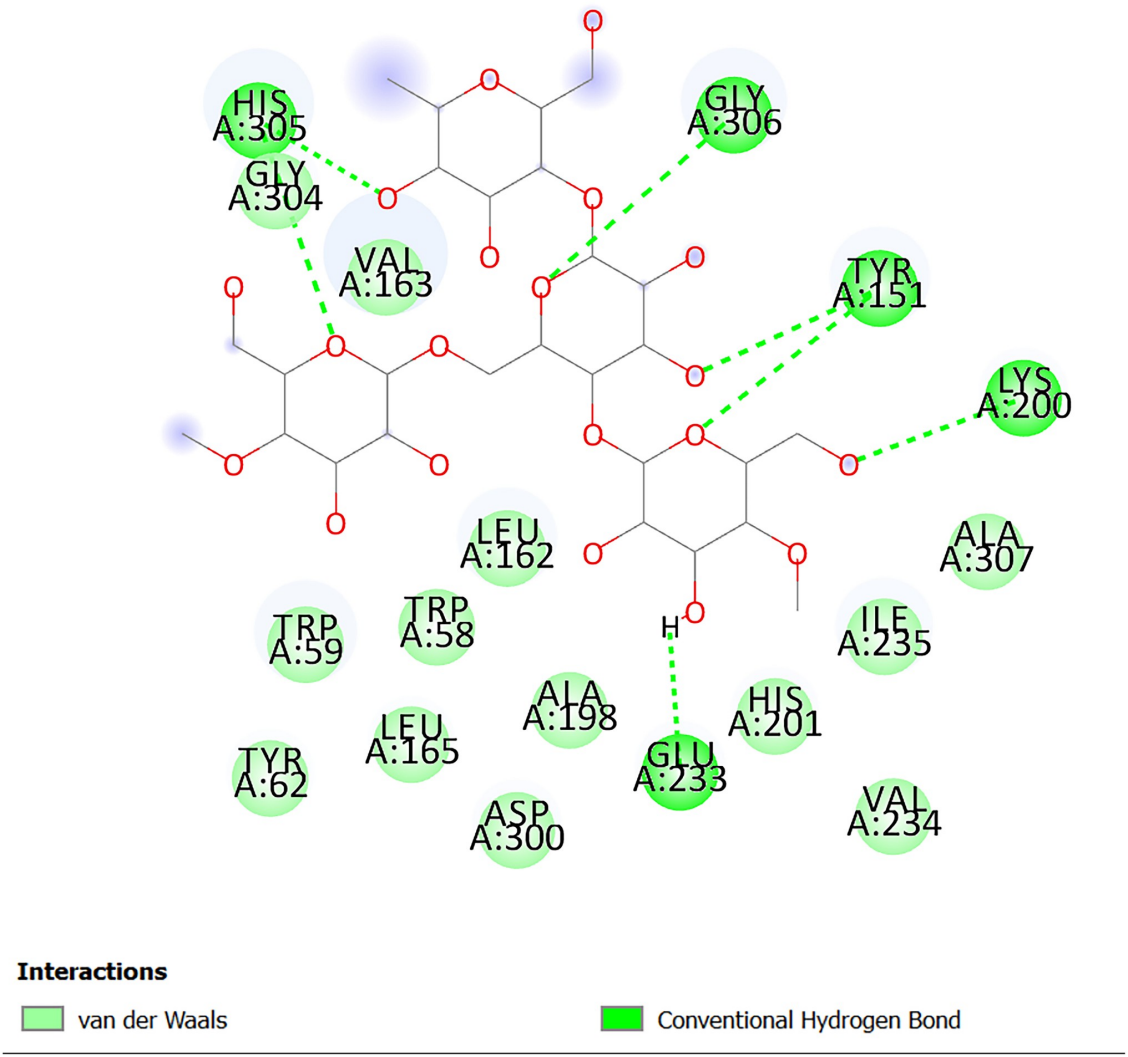
2D diagram of interactions between corn starch and 1OSE.

In addition, substrate (starch) was docked into the corresponding protein to analyse its ligand-target interactions and how they differ from the known inhibitor. According to the results, all four classes of interactions mentioned earlier present (Fig 3). However, only one residue formed an H-bond for each of these classes (only Glu233 can be found under the category of H-bond with acidic residues) whereas there are at least two residues involved in each interaction for acarbose (e.g. Glu233 and Asp300). Amongst these residues, three carboxyl groups Glu233, Asp300 and Asp197 are crucial for enzymatic activity and constitute the 3^rd^ subsite, in which Glu233 plays a crucial role in the first stage of the catalytic process as the general acid and a proton donor [22]. Therefore, acarbose serves as an effective inhibitor by preserving the key types of H-bonding and ring-ring interactions required for effective binding of ligands to the active site, and by direct binding to key residues such as Glu233, which normally functions as a nucleophilic attack moiety [22]. Additionally, it also forms additional supporting H-bonding interactions which more firmly anchors the inhibitor within the active site to prevent the entry of the endogenous substrate.

**Fig 4:**
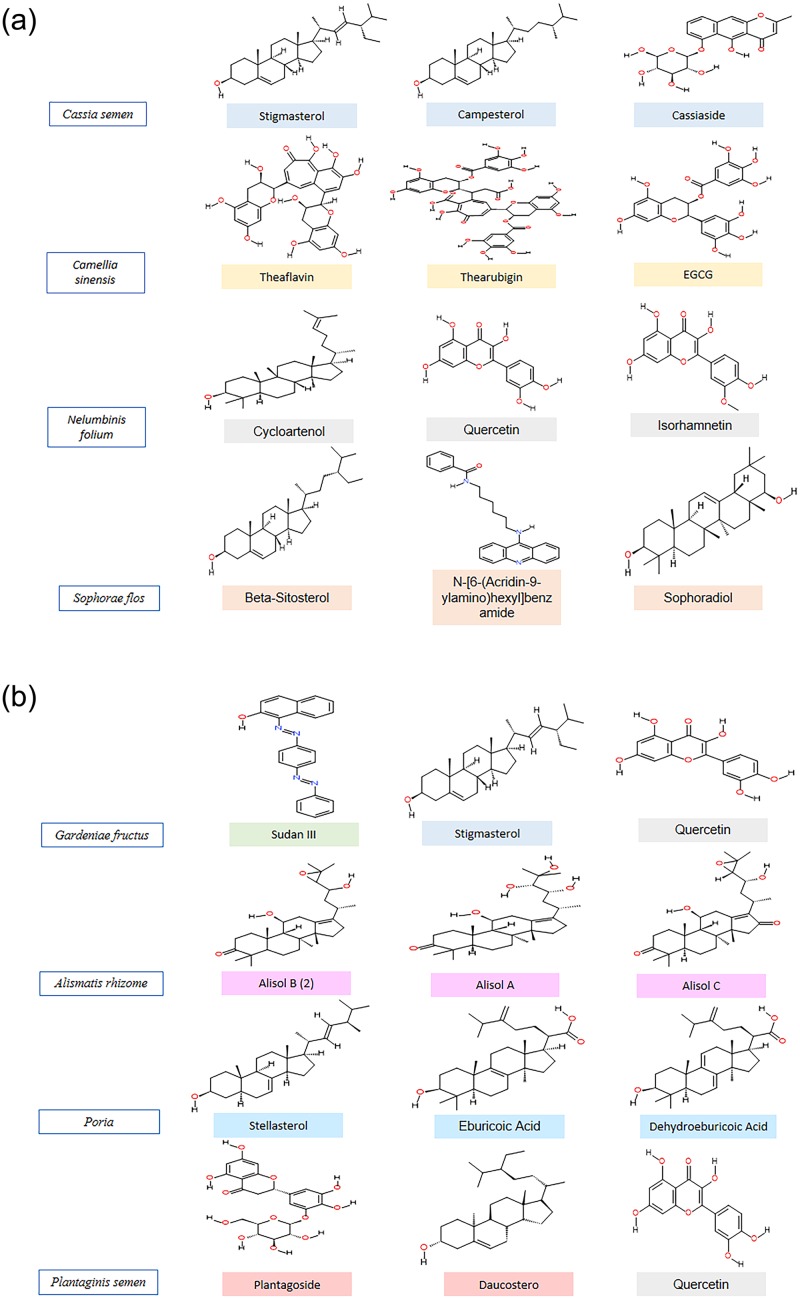
a-b. Chemical structures of selected compounds.

Subsequently, 149 main compounds from the eight herbs present in RCM-107 were docked with the target protein (PDB ID: 1OSE). Details for the top 3 highest binding affinity ligands for each herb (**1**. Stigmasterol; **2**. Campesterol; **3**. Cassiaside; **4**. Theaflavin; **5**. Thearubigin; **6**. EGCG; **7**. Cycloartenol; **8**. Quercetin; **9**. Isorhamnetin; **10**. Beta-sitosterol; **11**. N-[6-(acridin-9-ylamino) hexyl] benzamide; **12**. Sophoradiol; **13**. Sudan III; **14.** Alisol B; **15.** Alisol A; **16.** Alisol C; **17.** Stellasterol; **18.** Eburicoic acid; **19.** Dehydroeburicoic acid; **20.** Plantagoside; **21.** Daucosterol_qt, in which compound **1–3** are extracted from *Cassia semen*; **4–6** are from *Camellia sinensis*; **7–9** are from *Nelumbinis folium*; **10–12** are from *Sophorae flos*; **1, 8, 13** are from *Gardeniae fructus*; **14–16** are from *Alismatis rhizome*; **17–19** are from *Poria*; **8, 20–21** are from *Plantaginis semen*), including their chemical structures, active site residue interactions and predicted binding energy values, are presented in Table 1 and Fig 4. According to our results, docking energies of 57% of the compounds were superior to the known amylase inhibitor acarbose (-8.4 kcal/mol). In particular, compound **14** (alisol B, -10.7 kcal/mol) and **20** (plantagoside, -10.5 kcal/mol) exhibit the highest binding affinity, with both having substantially greater affinity values than acarbose, and are proposed to be effective inhibitors of PPA.

**Fig 5:**
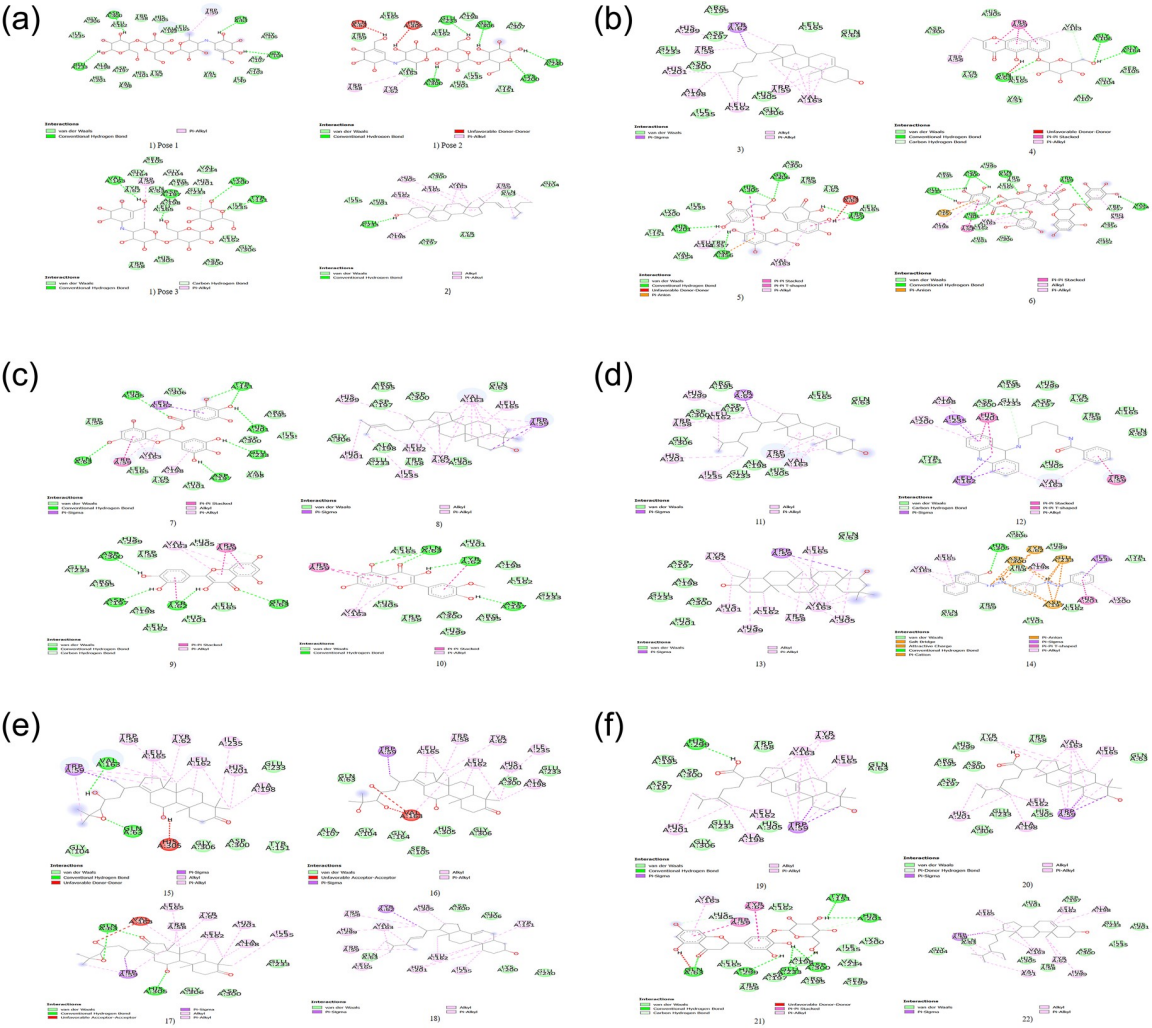
**a.** 2D diagram of top 3 predicted ligands from each herb with 1OSE. Hydrogen bonds (green circles with traced lines), van der Waals forces of attraction (light green circles), Pi-Alkyl interactions (pink circles with traced lines), Pi-Sigma interaction (purple circles with traced lines). 1) Acarbose (3 binding poses); 2). Stigmasterol. **b.** 3) Campesterol; 4) Cassiaside; 5) Theaflavin; 6) Thearubigin. **c.** 7) EGCG; 8) Cycloartenol; 9) Quercetin; 10) Isorhamnetin. **d.** 11) Beta-sitosterol; 12) N-[6-(acridin-9-ylamino) hexyl] benzamide; 13) Sophoradiol; 14) Sudan III. **e.** 15) Alisol B; 16) Alisol A. 17) Alisol C; 18) Stellasterol. **f.** 19) Eburicoic acid; 20) Dehydroeburicoic acid; 21) plantagoside; 22) Daucostero_qt.

In addition to the predicted binding affinity values, a qualitative inspection of the docked site can improve the accuracy and success rate of predicting inhibitor efficacy as Autodock was reported to have a typical error of ±2 kcal/mol [39]. In particular, we propose that ligands which share similar patterns of inter-residue interactions as those of the positive control inhibitor, acarbose, may also serve as potential effective inhibitors of PPA. The predicted docking interactions between the selected highest binding affinity ligands (**1**–**21** as well as acarbose) with alpha-amylase are presented in Fig 5a–5f.

According to the results obtained from the molecular docking, compounds **5**, **6** (extracted from *Camellia sinensis*) and **20** (obtained from *Plantaginis semen*) had more common residues with acarbose than other included ligands (in contrast, despite exhibiting the highest predicted binding affinity, compound **14** exhibited few common interactions with acarbose). Both compounds **6** and **20** displayed additional H-bonds for two main classes of interactions. Firstly, H-bonds with acidic residues Asp300 and Glu233 (compound **6** and **20**). Secondly, H-bonds with basic residues His305 and His201 (compound **6**) while compound **20** formed H-bonds with basic residues His299 and His201. Compound **5** did present H-bonds with a pair of acidic residues Glu233, Asp300 but only with one basic residue His305. In addition, there was only one aromatic ring-ring interaction with Trp59 for compound **5**. All three compounds had no H-bonds formed with the backbone of Gly residues, which however presented in the positive control acarbose. This may explain the role of RCM-107 and the relevant individual herbs in inhibiting amylase activities and why they are not as potent as acarbose as observed in this study.

**Fig 6:**
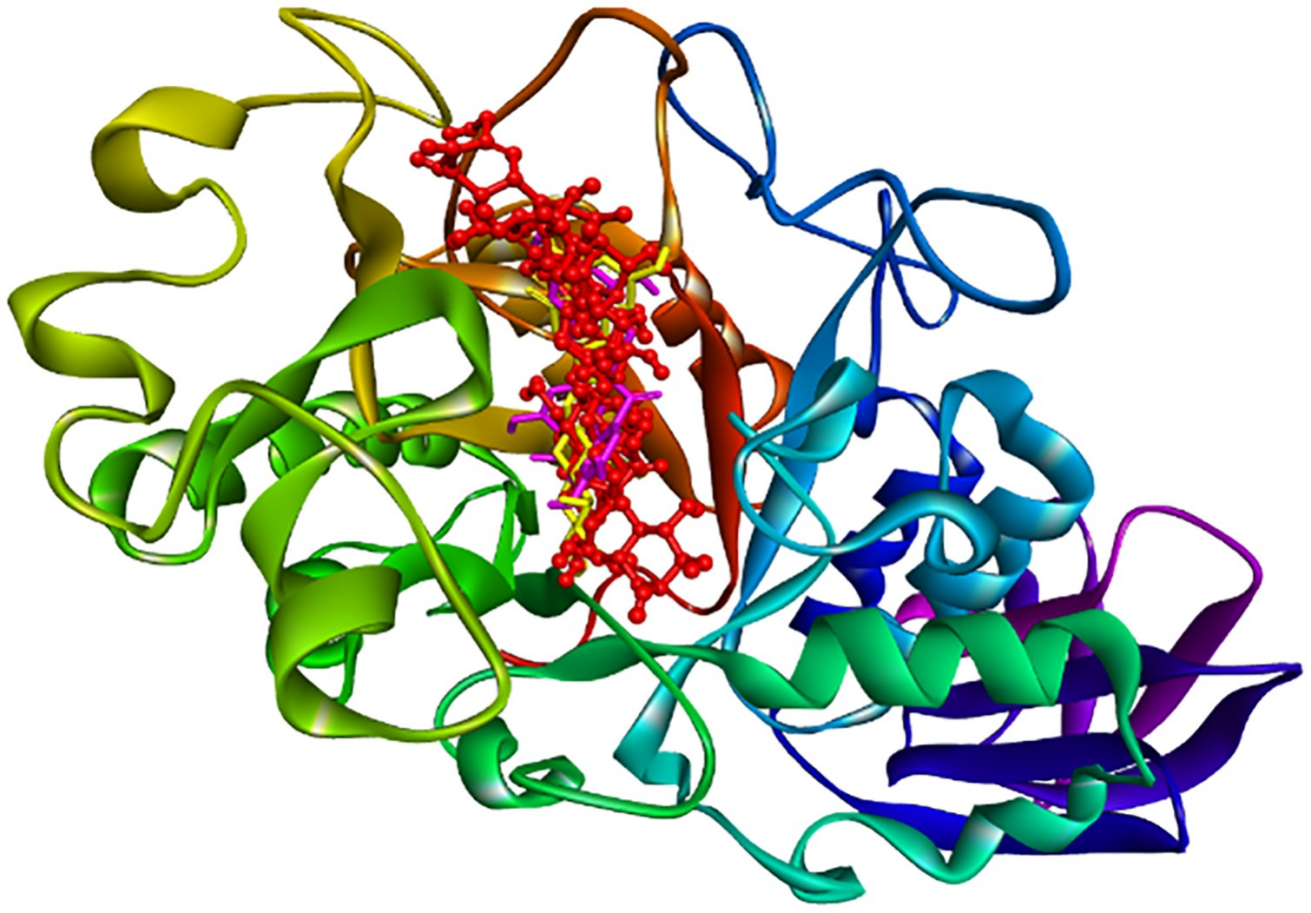
3D interactions between predicted leading compounds EGCG (pink), plantagoside (yellow), the known inhibitor acarbose (red) and 1OSE.

On the other hand, both *Poria* and *Alismatis rhizome* are unlikely to be effective amylase inhibitors as their top-ranked compounds formed no or a minimal number of common H-bonding residues with acarbose, which is consistent with the present inhibition results. Therefore, the corresponding residues (Glu233, Gln63, His305, Asp300 and Tyr151) can be considered as markers of important areas in the ligand-binding site. In addition, compounds **6** and **20** are more likely to act as the key compounds from RCM-107 for amylase inhibition (Fig 6).

Apart from H-bonding, other types of interactions such as van der Waals, Pi-Alkyl and Pi-Sigma interactions are also crucial to study the interactions between potential inhibitors and the enzyme PPA. Compounds **1**, **3**, **4**, **5**, **6**, **8**, **9**, **11**, **13**, **14**, **18**, **19, 20** formed multiple hydrophobic interactions with residues like Trp59, Val163, Leu162, Tyr62 and His 201, out of which Trp 59 and Tyr62 also formed this type of interaction with the known inhibitor acarbose. This may indicate an important relationship to the inhibitory mechanism for PPA (Fig 5a–5e). Although compounds **2**, **7**, **10**, **12**, **15, 16**, **17**, **21** didn’t form H-bonds with any amino acid residues, they showed Pi interactions with residues such as Tyr62, Trp58, Trp69, Val163, Leu162 and Leu165 (Fig 5a–5f).

Therefore, residues that form multiple non-polar interactions with the ligands, which are mediated via van der Waals contacts between the respective residues and moieties on the bound ligands, are proposed to make greater contributions to the overall non-polar interactions between the ligands and receptor, and therefore may be especially prone to cause disruption of binding to ligands if they are mutated to polar residues. Mutagenesis of these residues may be especially valuable, as they will be more likely to yield results which help validate our present predictions.

In summary, the highest-ranking compound is **alisol B** while the inter-residue contact analysis showed **EGCG** and **plantagoside** shared the most common residues with the known inhibitor and presented similar interactions, suggesting they may be responsible for the amylase inhibitory action of the RCM-107 (Table 1).

## Conclusion

The present work examined the alpha-amylase inhibition of the herbal formula RCM-107 and its constituent herbs and showed that the RCM-107 can be applied as an effective α-amylase inhibitor, which acts in a dose-dependent manner. Marginal (though not statistically significant) synergistic effects were observed for the RCM-107 formula when compared to the eight single herbs. However, the lack of observable significant synergistic effects may be related to the differences between the ratio of some individual herbs in RCM-107 and the use of equal concentrations of each herb in the present inhibition assay studies. We propose that RCM-107 can be optimized via increasing the proportion of *Gardeniae fructus* and *Nelumbinis folium* in the herbal mixture as those two individual herbs displayed higher amylase inhibition activities than the others. Also, alisol B, EGCG and plantagoside are proposed as the key active compounds that may play important roles in the α-amylase inhibition action of the RCM-107. Further validations such as site-directed mutagenesis or structure-activity relationships (quantitative predictions) are required to be carried out in future studies. In addition, bioassays that investigate the dose-dependent response of the individual components from RCM-107 to PPA will need to be conducted in the near future. The effects of our predicted key PPA inhibition compounds, as well as their toxicities, will need to be experimentally verified. Future experimental efforts should also involve examining the possible synergistic effects between pairs (or more) of individual molecular components of RCM-107. The results from such cross-reaction experiments will also help guide computational studies, eventually enabling rational design of RCM-107-based therapies with multiple compounds, exploiting their possible synergistic effects.

## Supporting information

S1 File (PDF)

S2 File (PDF)

S3 File (PDF)

S4 File (PDF)

S5 File (PDF)

S6 File (PDF)

S7 File (PDF)

S1 Quality assessments (PDF)

S1 Protocol Alpha-amylase assay protocol. (DOCX)

S1 Dataset Alpha-amylase assay dataset. (XLSX)

## References

[1] Jastreboff AM, Kotz CM, Kahan S, Kelly AS, Heymsfield SB. Obesity as a disease: The obesity society 2018 position statement. Obesity. 2019;27(1): 7–9. 10.1002/oby.22378 . 30569641

[2] Yan X, Zhang M, Wu T, Dai SD, Xu JL, Zhou ZK. The anti-obesity effect of green tea polysaccharides, polyphenols and caffeine in rats fed with a high-fat diet. Food Funct. 2015;6(1): 296–303. 10.1039/c4fo00970c 25431018

[3] Piche ME, Poirier P, Lemieux I, Despres JP. Overview of epidemiology and contribution of obesity and body fat distribution to cardiovascular disease: An update. Prog Cardiovasc Dis. 2018;61(2): 103–13. 10.1016/j.pcad.2018.06.004 . 29964067

[4] WHO. 10 Facts on obesity: WHO; 2019 [cited 2019 18 January]. https://www.who.int/features/factfiles/obesity/en/

[5] Tucci SA, Boyland EJ, Halford JC. The role of lipid and carbohydrate digestive enzyme inhibitors in the management of obesity: A review of current and emerging therapeutic agents. Diabetes Metab Syndr Obes. 2010;3: 125 10.2147/dmsott.s7005 21437083 PMC3047983

[6] Marrelli M, Loizzo MR, Nicoletti M, Menichini F, Conforti F. Inhibition of key enzymes linked to obesity by preparations from Mediterranean dietary plants: Effects on α-amylase and pancreatic lipase activities. Plant Foods Hum Nutr. 2013;68(4): 340–6. 10.1007/s11130-013-0390-9 24122547

[7] Luo S, Lenon GB, Gill H, Yuen H, Yang AWH, Hung A, et al Do the natural chemical compounds Interact with the same targets of current pharmacotherapy for weight management? A Review. Curr Drug Targets. 2019;20(4): 399–411. 10.2174/1389450119666180830125958 . 30173643

[8] Barrett ML, Udani JK. A proprietary alpha-amylase inhibitor from white bean (Phaseolus vulgaris): A review of clinical studies on weight loss and glycemic control. Nutrition. 2011;10(1): 24. 10.1186/1475-2891-10-24 PMC3071778 21414227

[9] Moyers SB. Medications as adjunct therapy for weight loss: Approved and off-label agents in use. J Am Diet Assoc. 2005;105(6): 948–59. 10.1016/j.jada.2005.03.010 . 15942547

[10] Udani J, S BB. Blocking carbohydrate absorption and weight loss: A clinical trial using a proprietary fractionated white bean extract. Altern Ther Health Med. 2007;13(4): 32–9. 17658120

[11] Xu X, Li F, Zhang X, Li P, Zhang X, Wu Z, et al In vitro synergistic antioxidant activity and identification of antioxidant components from Astragalus membranaceus and Paeonia lactiflora. PLoS One. 2014;9(5): e96780 10.1371/journal.pone.0096780 . 24816851 PMC4016014

[12] Li X, Xu X, Wang J, Yu H, Wang X, Yang H, et al A system-level investigation into the mechanisms of chinese traditional medicine: Compound Danshen formula for cardiovascular disease treatment. PLoS One. 2012;7(9): e43918 10.1371/journal.pone.0043918 . 22962593 PMC3433480

[13] Yue G, Wei J, Qian X, Yu L, Zou Z, Guan W, et al Synergistic anticancer effects of polyphyllin I and evodiamine on freshly-removed human gastric tumors. PLoS One. 2013;8(6): e65164 10.1371/journal.pone.0065164 . 23762305 PMC3676398

[14] Tao W, Xu X, Wang X, Li B, Wang Y, Li Y, et al Network pharmacology-based prediction of the active ingredients and potential targets of Chinese herbal Radix Curcumae formula for application to cardiovascular disease. J Ethnopharmacol. 2013;145(1): 1–10. 10.1016/j.jep.2012.09.051 . 23142198

[15] Park J, Jeon YD, Kim HL, Lim H, Jung Y, Youn DH, et al Interaction of Veratrum nigrum with Panax ginseng against obesity: A sang-ban relationship. eCAM. 2013;2013: e732126 10.1155/2013/732126 . 24073007 PMC3773901

[16] Wang X, Xu X, Tao W, Li Y, Wang Y, Yang L. A systems biology approach to uncovering pharmacological synergy in herbal medicines with applications to cardiovascular disease. eCAM. 2012;2012: e519031 10.1155/2012/519031 . 23243453 PMC3518963

[17] Yang WJ, Li DP, Li JK, Li MH, Chen YL, Zhang PZ. Synergistic antioxidant activities of eight traditional Chinese herb pairs. Biol Pharm Bull. 2009;32(6): 1021–6. 10.1248/bpb.32.1021 19483308

[18] Zhou X, Seto SW, Chang D, Kiat H, Razmovski Naumovski V, Chan K, et al Synergistic effects of Chinese herbal medicine: A comprehensive review of methodology and current research. Front Pharmacol. 2016;7: 201 10.3389/fphar.2016.00201 . 27462269 PMC4940614

[19] Lenon GB, Li KX, Chang YH, Yang AW, Da Costa C, Li CG, et al Efficacy and Safety of a Chinese herbal medicine formula (RCM-104) in the management of simple obesity: A randomized, placebo-controlled clinical trial. eCAM. 2012;2012: 435702 10.1155/2012/435702 . 22550541 PMC3328918

[20] Bensky D, Clavey S, Gamble A, Stö E, Bensky LL. Chinese Herbal Medicine: Materia Medica. 3rd ed Seattle, WA: Eastland Press, Inc; 2004.

[21] Luo S, Gill H, Dias DA, Li M, Hung A, Nguyen LT, et al The inhibitory effects of an eight-herb formula (RCM-107) on pancreatic lipase: enzymatic, HPTLC profiling and in silico approaches. Heliyon. 2019;5(9): e02453 10.1016/j.heliyon.2019.e02453 . 31538117 PMC6745409

[22] Qian MX, Haser R, Buisson G, Duee E, Payan F. The active center of a mammalian .alpha.-amylase. structure of the complex of a pancreatic .alpha.-amylase with a carbohydrate inhibitor refined to 2.2.ANG. resolution. Biochemistry. 2002;33(20): 6284–94. 10.1021/bi00186a031 8193143

[23] Xiao W, Li S, Wang S, Ho CT. Chemistry and bioactivity of Gardenia jasminoides. J Food Drug Anal. 2017;25(1): 43–61. 10.1016/j.jfda.2016.11.005 . 28911543 PMC9333430

[24] Wang F, Xiong ZY, Li P, Yang H, Gao W, Li HJ. From chemical consistency to effective consistency in precise quality discrimination of Sophora flower-bud and Sophora flower: Discovering efficacy-associated markers by fingerprint-activity relationship modeling. J Pharm Biomed Anal. 2017;132: 7–16. 10.1016/j.jpba.2016.09.042 . 27693758

[25] Turkozu D, Tek NA. A minireview of effects of green tea on energy expenditure. Crit Rev Food Sci Nutr. 2017;57(2): 254–8. 10.1080/10408398.2014.986672 . 26091183

[26] Sharma BR, Gautam LN, Adhikari D, Karki R. A comprehensive review on chemical profiling of Nelumbo Nucifera: Potential for drug development. Phytother Res. 2017;31(1): 3–26. 10.1002/ptr.5732 . 27667670

[27] Dong X, Fu J, Yin X, Yang C, Zhang X, Wang W, et al Cassiae semen: A review of its phytochemistry and pharmacology. Mol Med Rep. 2017;16(3): 2331–46. 10.3892/mmr.2017.6880 . 28677746 PMC5547955

[28] Wu LF, Wang KF, Mao X, Liang WY, Chen WJ, Li S, et al Screening and analysis of the potential bioactive components of Poria cocos (Schw.) Wolf by HPLC and HPLC-MS(n) with the aid of chemometrics. Molecules. 2016;21(2). 10.3390/molecules21020227 . 26901179 PMC6274397

[29] Paudel KR, Panth N. Phytochemical profile and biological activity of Nelumbo nucifera. eCAM. 2015;2015: e789124 10.1155/2015/789124 . 27057194 PMC4710907

[30] Han Y, Wen J, Zhou T, Fan G. Chemical fingerprinting of Gardenia jasminoides Ellis by HPLC-DAD-ESI-MS combined with chemometrics methods. Food Chem. 2015;188: 648–57. 10.1016/j.foodchem.2015.05.039 . 26041243

[31] Rios JL. Chemical constituents and pharmacological properties of Poria cocos. Planta Med. 2011;77(7): 681–91. 10.1055/s-0030-1270823 . 21347995

[32] Geng F, Yang L, Chou GX, Wang ZT. Bioguided isolation of angiotensin-converting enzyme inhibitors from the seeds of Plantago asiatica L. Phytother Res. 2009: 1088–94. 19998322 10.1002/ptr.3071

[33] Perva-Uzunalić A, Škerget M, Knez Ž, Weinreich B, Otto F, Grüner S. Extraction of active ingredients from green tea (Camellia sinensis): Extraction efficiency of major catechins and caffeine. Food Chem. 2006;96(4): 597–605. 10.1016/j.foodchem.2005.03.015

[34] Wagner H, Bauer R, Melchart D, Xiao PG, Staudinger A. Chromatographic Fingerprint Analysis of Herbal Medicines Thin-Layer and High Performance Liquid Chromatography of Chinese Drugs. 2nd ed Vienna: Springer Vienna; 2011.

[35] Sun GL, Zhao Q, Dong XN, Wang YP. Pharmacological and chemical composition study of Huai Hua. J Tradit Chin Veter Med. 2009;28(6): 24–7.

[36] Yilmazer-Musa M, Griffith AM, Michels AJ, Schneider E, Frei B. Grape seed and tea extracts and catechin 3-gallates are potent inhibitors of alpha-amylase and alpha-glucosidase activity. J Agric Food Chem. 2012;60(36): 8924–9. 10.1021/jf301147n . 22697360 PMC4356113

[37] Huang C, Zheng C, Li Y, Wang Y, Lu A, Yang L. Systems pharmacology in drug discovery and therapeutic insight for herbal medicines. Brief Bioinform. 2014;15(5): 710–33. 10.1093/bib/bbt035 . 23736100

[38] Gilles C, Astier P, Marchis M, G., Cambillau C, Payan F. Crystal structure of pig pancreatic α-amylase isoenzyme II, in complex with the carbohydrate inhibitor acarbose. Eur J Biochem. 1996;238(2): 561–9. 10.1111/j.1432-1033.1996.0561z.x 8681972

[39] Manoj K, Anuj S, Anuradha D, G V. K. In silico docking studies of bioactive natural plant products as putative DHFR antagonists. Med Chem Res. 2014;23: 810–7. 10.1007/s00044-013-0654-9

