# Supplementary material for: Inhibitory effect of a weight-loss Chinese herbal formula RCM-107 on pancreatic α-amylase activity: Enzymatic and in silico approaches
Source: PLoS One. 2020 Apr 29;15(4):e0231815. doi: 10.1371/journal.pone.0231815 (PMC7190128; doi:10.1371/journal.pone.0231815)
Supplement: S1 File — (PDF) [file pone.0231815.s001.pdf]

培力（南寧）藥業有限公司檢測中心 **PuraPharm (Nanning) Pharmaceuticals Co.,Ltd. Testing Laboratory**
中國廣西南寧市高新技術開發區科園大道 46 號
No.46, Ke Yuan Road,Nanning New & High-tech Industrial Development Zone, Guangxi,China.

對“澤瀉配方顆粒”之樣品之分析報告

Report on the submitted sample identified by the client - Ze Xie

樣品名稱 Product Description	澤瀉配方顆粒 Ze Xie
樣品編號 Product Code	1015
樣品規格 Product Specification	200 克/瓶 g/ bottle
批號 Batch No.	A1600004
有效期至(年/月/日)Expiry Date（yyyy/mm/dd）	2019/01/31
本批數量 Quantity Produced	5326 瓶 bottles
樣品收到時狀態 Sample Receiving Condition	室溫下存放於密封塑膠樽原來包裝中<br>In sealed bottle of the original package under ambient condition.
製造商 Manufacturer	生產部 Production Department
委託檢驗單位 Inspected Entity	生產部 Production Department
來源地 Region of Origin	生產部 Production Department
目的地 Region of Destination	培力（南寧）藥業有限公司檢測中心<br>PuraPharm (Nanning) Pharmaceuticals Co.,Ltd. Testing Laboratory
檢驗日期(年/月/日)Testing Period（yyyy/mm/dd）	2016/02/03 - 2016/02/09

測試項目、分析方法及分析結果 Test Requested, Test Method and Test Results

請參考續頁。Please refer to the following page(s)

****

培力（南寧）藥業有限公司檢測中心代表簽名

Signed for and on behalf of PuraPharm (Nanning) Pharmaceuticals Co.,Ltd. Testing Laboratory

測試項目及分析方法 Test Requested and Test Method

測試項目 Test Items	參考方法 Reference Method
1. 性狀 Appearance	中國藥典, 2015 年版、第四部、通則 0104<br>The Pharmacopoeia of the People's Republic of China 2015, Vol.4, General chapter 0104
2. 水分 Determination of Water	中國藥典, 2015 年版、第四部、通則 0832<br>The Pharmacopoeia of the People's Republic of China 2015, Vol.4, General chapter 0832
3. 粒度 Size of Granule	中國藥典, 2015 年版、第四部、通則 0982<br>The Pharmacopoeia of the People's Republic of China 2015, Vol.4, General chapter 0982
4. 溶化性<br>Determination of Dispersibility	中國藥典, 2015 年版、第四部、通則 0104<br>The Pharmacopoeia of the People's Republic of China 2015, Vol.4, General chapter 0104
5. 裝量 Capacity	中國藥典, 2015 年版、第四部、通則 0942<br>The Pharmacopoeia of the People's Republic of China 2015, Vol.4, General chapter 0942
6. 重金屬及有害元素<br>Heavy Metals and Toxic Elements	中國藥典, 2015 年版、第四部、通則 2321；電感耦合等離子體質譜法；<br>The Pharmacopoeia of the People's Republic of China 2015, Vol.4, General chapter 2321；ICP-MS
7. 農藥殘留 Pesticides Residues	香港中醫藥管理委員會《中成藥註冊申請手冊》<br>Chinese Medicine Council of Hong Kong《Application Form: Registration of proprietary Chinese medicines》
8. 微生物限度 Microbial Limit	中國藥典, 2015 年版、第四部、通則 1105、1106、1107<br>The Pharmacopoeia of the People's Republic of China 2015, Vol.4, General chapter 1105，1106，1107
- 需氧菌總數 TAMC (Total Aerobic Microbial Count)	
- 霉菌和酵母菌總數 TYMC（Total Yeast and Mold Microbial Count)	
- 大腸埃希菌 Escherichia Coli	

****

編制 Report compiled by：[signature]　　審核 Reviewed by：[signature]

分析結果 Test Results

測試項目 Test Items	品質標準 Quality Specification	澤瀉配方顆粒 Ze Xie
1. 性狀 Appearance	本品為黃色至淡黃棕色顆粒，氣微，味酸、微苦。The product is yellow to light yellowish brown granule, slight odour, sour and slight bitter taste.	符合規定 Conform
2. 水分 Determination of Water	≤6.5% (W/W)	4.4%<br>符合規定 Conform
3. 粒度 Size of Granule	不能通過一號篩和能通過五號篩的顆粒和粉末總和不得超過 15% Sum of weight of granules that cannot pass through sieve No.1 and weight of powder that can pass through sieve No.5≤15%	4%<br>符合規定 Conform
4. 溶化性<br>Determination of Dispersibility	應符合規定 Shall meet the requirement	符合規定 Conform
5. 裝量 Capacity	應符合規定 Shall meet the requirement	符合規定 Conform
6. 重金屬及有害元素#<br>Heavy Metals and Toxic Elements		
- 銅 Copper (Cu)	不得過 150.00mg/kg<br>Cu≤150.00 mg/kg	4.514mg/kg<br>符合規定 Conform
- 砷 Arsenic (As)	不得過 41.67mg/kg 或 1500μg/日<br>As≤41.67mg/kg or 1,500μg/day	0.192mg/kg（6.91μg/日 day）<br>符合規定 Conform
- 鎘 Cadmium (Cd)	不得過 97.22mg/kg 或 3500μg/劑<br>Cd≤97.22mg/kg or 3,500μg/dose	0.031mg/kg（1.12μg/劑 dose）<br>符合規定 Conform
- 鉛 Lead (Pb)	不得過 4.97mg/kg 或 179μg/日<br>Pb≤4.97mg/kg or 179μg/day	0.025mg/kg（0.90μg/日 day）<br>符合規定 Conform
- 汞 Mercury (Hg)	不得過 1.00mg/kg 或 36μg/日<br>Hg≤1.00mg/kg or 36μg/day	＜0.001mg/kg（＜0.04μg/日 day）<br>符合規定 Conform
7. 農藥殘留#<br>Pesticides Residues		
- 艾氏劑及狄氏劑<br>Aldrin & Dieldrin	艾氏劑及狄氏劑兩者之和≤0.05 毫克/千克<br>Sum of aldrin & dieldrin≤0.05 mg/kg<br>（檢出限艾氏劑 1.77μg/kg,狄氏劑 2.43μg/kg<br>LOD aldrin 1.77μg/kg, dieldrin 2.43μg/kg）	未檢出 Not detected<br>符合規定 Conform
- 氯丹 Chlordane	順式-,反式及氧化氯丹之和≤0.05 毫克/千克<br>Sum of cis-,trans- & oxy-chlordane≤0.05 mg/kg<br>（檢出限順式氯丹 2.05μg/kg, 反式氯丹 1.81μg/kg, 氧化氯丹 2.21μg/kg<br>LOD cis- chlordane 2.05μg/kg, trans- chlordane 1.81μg/kg, oxy-chlordane 2.21μg/kg）	未檢出 Not detected<br>符合規定 Conform
- 滴滴涕 DDT	4,4'-滴滴涕, 2,4'-滴滴涕, 4,4'-滴滴伊及 4,4'-滴滴滴之和≤1.0 毫克/千克<br>Sum of *p, p*'-DDT, *o,p*'-DDT, *p,p*'-DDE, *p,p*'-TDE ≤1.0 mg/kg<br>（檢出限 4,4'-滴滴涕 3.73μg/kg, 2,4'-滴滴涕 4.48μg/kg, 4,4'-滴滴伊 2.29μg/kg, 4,4'-滴滴滴 2.88μg/kg<br>LOD *p, p*'-DDT 3.73μg/kg, *o,p*'-DDT 4.48μg/kg, *p,p*'-DDE 2.29μg/kg, *p,p*'-TDE 2.88μg/kg）	未檢出 Not detected<br>符合規定 Conform
- 異狄氏劑 Endrin	≤0.05 mg/kg<br>（檢出限 LOD 3.78μg/kg）	未檢出 Not detected<br>符合規定 Conform

****

編制 Report compiled by：[signature]　　審核 Reviewed by：[signature]

培力（南寧）藥業有限公司檢測中心 PuraPharm (Nanning) Pharmaceuticals Co.,Ltd. Testing Laboratory
郵編(Postal No.)：530007　電話(Tel)：0771-3218026　傳真(Fax)：0771-3216602

- 七氯 Heptachlor	七氯及環氧七氯之和≤0.05 毫克/千克<br>Sum of heptachlor & heptachlor epoxide≤0.05 mg/kg<br>（檢出限七氯 1.78μg/kg、環氧七氯 1.97μg/kg<br>LOD heptachlor 1.78μg/kg, heptachlor epoxide 1.97μg/kg）	未檢出 Not detected<br>符合規定 Conform
- 六氯苯 Hexachlorobenzene	≤0.1 mg/kg<br>（檢出限 LOD 2.16μg/kg）	未檢出 Not detected<br>符合規定 Conform
- 六六六 Hexachlorocyclohexane	α-,β-及 δ-異構體之和≤0.3 毫克/千克<br>Sum of α-,β-,δ- isomers≤0.3 mg/kg<br>（檢出限 α-六六六 1.61μg/kg、β-六六六 2.96μg/kg、δ-六六六 1.43μg/kg<br>LOD α- HCH 1.61μg/kg, β- HCH 2.96μg/kg, δ- HCH 1.43μg/kg）	未檢出 Not detected<br>符合規定 Conform
- 林丹 Lindane	≤0.6 mg/kg<br>（檢出限 LOD 1.56μg/kg）	未檢出 Not detected<br>符合規定 Conform
- 五氯硝基苯 Quintozene	五氯硝基苯，五氯苯胺及甲基五氯硫基苯之和≤1.0 毫克/千克<br>Sum of quintozene, pentachloroaniline and methyl pentachlorophenyl sulphide≤1.0 mg/kg<br>（檢出限五氯硝基苯 1.92μg/kg、五氯苯胺 1.59μg/kg、甲基五氯硫基苯 1.33μg/kg<br>LOD quintozene 1.92μg/kg , pentachloroaniline 1.59μg/kg , methyl pentachlorophenyl sulphide 1.33μg/kg）	未檢出 Not detected<br>符合規定 Conform
8. 微生物限度#<br>Microbial Limit		
- 需氧菌總數 TAMC (Total Aerobic Microbial Count)	≤500cfu/g	<10cfu/g<br>符合規定 Conform
-霉菌和酵母菌總數 TYMC (Total Yeast and Mold Microbial Count)	≤100cfu/g	<10cfu/g<br>符合規定 Conform
- 大腸埃希菌 Escherichia Coli	不得檢出/克 Not detected/g	未檢出/克 Not detected/g<br>符合規定 Conform

****

编制 Report compiled by：罗昌将 審核 Reviewed by：周继行

培力（南寧）藥業有限公司檢測中心 PuraPharm (Nanning) Pharmaceuticals Co.,Ltd. Testing Laboratory

郵編(Postal No.)：530007 電話(Tel)：0771-3218026 傳真(Fax)：0771-3216602

註：Remarks

1、 報告無"檢驗/檢測報告專用章" 和缺騎縫章無效。

Test report without the stamp of "For Test/Test Report Only" is not authorized.

2、 複製的報告未重新加蓋"檢驗/檢測報告專用章" 及騎縫章無效。

Certified copy of test reportwithout the stamp of"For Test/Test Report Only" is not authorized.

3、 報告無編制、審核及本中心代表簽字無效。

Test report must be authorized by the signature of the bodies of preparation, verification and representative of the testing centre.

4、 報告塗改、缺頁無效。

Test report with modification or lacking of page(s) is not authorized.

5、 對報告若有異議，請於收到報告之日起十五日內向檢驗單位提出書面申訴，否則按認可檢驗報告處理。本中心異議受理電話 0771-3218026。

The client is aggrieved by the test result. A request for review shall state in writing the reasons relied upon and shall be made to the testing centre within 15 days after receipt of the authorized test report. No requirement will be accepted after the definite time. Inquiry hot line is 0771-3218026.

6、 送樣委託檢驗，樣品名稱為委託單位自報名稱，報告僅對來樣負責。部份複製檢驗/檢測報告無效。

Name of tested product is provided by the client. The report will refer only to the sample tested. Copy of partial of the test report is not authorized.

# - 其他微生物限度要求: Other requirements for Microbial Limit Test

1. 含動物類藥材（包括提取物）的中藥固體製劑，每 10g 不得檢出沙門菌。

TCM solid preparation containing animal raw materials (including extract): Absence of Salmonella (10g).

2. 含藥材原粉的中藥固體製劑，每 10g 不得檢出沙門菌；耐膽鹽革蘭陰性菌應小於 10^2cfu（1g）。

TCM solid preparation containing herbal raw powders: Absence of Salmonella (10g); Not more than 10^2cfu of Bile-tolerant gram-negative bacteria (1g).

*** End of Report 報告完 ***

培力（南寧）藥業有限公司檢測中心 PuraPharm (Nanning) Pharmaceuticals Co.,Ltd. Testing Laboratory
郵編(Postal No.)：530007 電話(Tel)：0771-3218026 傳真(Fax)：0771-3216602
