# Supplementary material for: Inhibitory effect of a weight-loss Chinese herbal formula RCM-107 on pancreatic α-amylase activity: Enzymatic and in silico approaches
Source: PLoS One. 2020 Apr 29;15(4):e0231815. doi: 10.1371/journal.pone.0231815 (PMC7190128; doi:10.1371/journal.pone.0231815)
Supplement: S2 File — (PDF) [file pone.0231815.s002.pdf]

培力（南寧）藥業有限公司檢測中心 PuraPharm (Nanning) Pharmaceuticals Co.,Ltd. Testing Laboratory

中國廣西南寧市高新技術開發區 No.46, Ke Yuan Road,Nanning New & High-tech

科園大道 46 號 Industrial Development Zone, Guangxi,China.

對“決明子配方顆粒”之樣品之分析報告

Report on the submitted sample identified by the client - Jue Ming Zi

樣品名稱 Product Description	決明子配方顆粒 Jue Ming Zi
樣品編號 Product Code	1161
樣品規格 Product Specification	200 克/瓶 g/ bottle
批號 Batch No.	A1500060
有效期至(年/月/日)Expiry Date（yyyy/mm/dd）	2018/02/05
本批數量 Quantity Produced	2333 瓶 bottles
樣品收到時狀態 Sample Receiving Condition	室溫下存放於密封塑膠樽原來包裝中<br>In sealed bottle of the original package under ambient condition.
製造商 Manufacturer	生產部 Production Department
委託檢驗單位 Inspected Entity	生產部 Production Department
來源地 Region of Origin	生產部 Production Department
目的地 Region of Destination	培力（南寧）藥業有限公司檢測中心<br>PuraPharm (Nanning) Pharmaceuticals Co.,Ltd. Testing Laboratory
檢驗日期(年/月/日)Testing Period（yyyy/mm/dd）	2015/02/09 - 2015/03/03

測試項目、分析方法及分析結果 Test Requested, Test Method and Test Results

請參考續頁。Please refer to the following page(s)

****

培力（南寧）藥業有限公司檢測中心代表簽名

Signed for and on behalf of PuraPharm (Nanning) Pharmaceuticals Co.,Ltd. Testing Laboratory

測試項目及分析方法 Test Requested and Test Method

測試項目 Test Items	参考方法 Reference Method
1. 性狀 Appearance	中國藥典, 2010 年版, 第一部, 附錄 I C<br>The Pharmacopoeia of the People's Republic of China 2010, Vol.1, Appendix I C
2. 水分 Determination of Water	中國藥典, 2010 年版, 第一部, 附錄Ⅸ H<br>The Pharmacopoeia of the People's Republic of China 2010, Vol.1, Appendix Ⅸ H
3. 粒度 Size of Granule	中國藥典, 2010 年版, 第一部, 附錄XI B<br>The Pharmacopoeia of the People's Republic of China 2010, Vol.1, Appendix XI B
4. 溶化性<br>Determination of Dispersibility	中國藥典, 2010 年版, 第一部, 附錄 I C<br>The Pharmacopoeia of the People's Republic of China 2010, Vol.1, Appendix I C
5. 裝量 Capacity	中國藥典, 2010 年版, 第一部, 附錄Ⅻ C<br>The Pharmacopoeia of the People's Republic of China 2010, Vol.1, AppendixⅫ C
6. 重金屬及有害元素<br>Heavy Metals and Toxic Elements	中國藥典, 2010 年版, 第一部, 附錄Ⅸ B；電感耦合等離子體質譜法；<br>The Pharmacopoeia of the People's Republic of China 2010, Vol.1, Appendix Ⅸ B；<br>ICP-MS
7. 農藥殘留 Pesticides Residues	香港中醫藥管理委員會《中成藥註冊申請手冊》<br>Chinese Medicine Council of Hong Kong《Application Form: Registration of proprietary Chinese medicines》
8. 微生物限度 Microbial Limit	中國藥典, 2010 年版, 第一部, 附錄 XIII C<br>The Pharmacopoeia of the People's Republic of China 2010, Vol.1, Appendix XIII C
- 細菌數 Microbial Count	
- 霉菌及酵母菌<br>Mould and Yeast Count	
- 大腸埃希菌 Escherichia Coli	

****

編制 Report compiled by：[illegible]　　審核 Reviewed by：[illegible]

分析結果 Test Results

測試項目 Test Items	品質標準 Quality Specification	決明子配方顆粒 Jue Ming Zi
1. 性狀 Appearance	本品為淺灰黃色至棕褐色顆粒，氣微，味淡，微苦。<br>The product is light greyish yellow to brown granule, slight odour, weak and slightly bitter taste.	符合規定 Conform
2. 水分 Determination of Water	≤6.0% (W/W)	3.5%<br>符合規定 Conform
3. 粒度 Size of Granule	不能通過一號篩和能通過五號篩的顆粒和粉末總和不得超過 15% Sum of weight of granules that cannot pass through sieve No.1 and weight of powder that can pass through sieve No.5≤15%	2%<br>符合規定 Conform
4. 溶化性<br>Determination of Dispersibility	應符合規定 Shall meet the requirement	符合規定 Conform
5. 裝量 Capacity	應符合規定 Shall meet the requirement	符合規定 Conform
6. 重金屬及有害元素#<br>Heavy Metals and Toxic Elements		
- 銅 Copper (Cu)	不得過 150.00mg/kg<br>Cu≤150.00 mg/kg	0.961mg/kg<br>符合規定 Conform
- 砷 Arsenic (As)	不得過 41.67mg/kg 或 1500μg/日<br>As≤41.67mg/kg or 1,500μg/day	0.028mg/kg（1.01μg/日 day）<br>符合規定 Conform
- 鎘 Cadmium (Cd)	不得過 97.22mg/kg 或 3500μg/劑<br>Cd≤97.22mg/kg or 3,500μg/dose	0.039mg/kg（1.40μg/劑 dose）<br>符合規定 Conform
- 鉛 Lead (Pb)	不得過 4.97mg/kg 或 179μg/日<br>Pb≤4.97mg/kg or 179μg/day	0.072mg/kg（2.59μg/日 day）<br>符合規定 Conform
- 汞 Mercury (Hg)	不得過 1.00mg/kg 或 36μg/日<br>Hg≤1.00mg/kg or 36μg/day	＜0.001mg/kg（＜0.04μg/日 day）<br>符合規定 Conform
7. 農藥殘留#<br>Pesticides Residues		
- 艾氏劑及狄氏劑<br>Aldrin & Dieldrin	艾氏劑及狄氏劑兩者之和≤0.05 毫克/千克<br>Sum of aldrin & dieldrin≤0.05 mg/kg	未檢出 Not detected<br>符合規定 Conform
- 氯丹 Chlordane	順式-,反式及氧化氯丹之和≤0.05 毫克/千克<br>Sum of cis-,trans- & oxychlordane≤0.05 mg/kg	未檢出 Not detected<br>符合規定 Conform
- 滴滴涕 DDT	4,4'-滴滴涕, 2,4'-滴滴涕, 4,4'-滴滴伊及 4,4'-滴滴滴之和≤1.0 毫克/千克<br>Sum of *p, p*'-DDT, *o,p*'-DDT, *p,p*'-DDE, *p,p*'-TDE ≤1.0 mg/kg	未檢出 Not detected<br>符合規定 Conform
- 異狄氏劑 Endrin	≤0.05 mg/kg	未檢出 Not detected<br>符合規定 Conform
- 七氯 Heptachlor	七氯及環氧七氯之和≤0.05 毫克/千克<br>Sum of heptachlor & heptachlor epoxide≤0.05 mg/kg	未檢出 Not detected<br>符合規定 Conform
- 六氯苯 Hexachlorobenzene	≤0.1 mg/kg	未檢出 Not detected<br>符合規定 Conform
- 六六六 Hexachlorocyclohexane	α-,β-及 δ-異構體之和≤0.3 毫克/千克<br>Sum of α-,β-,δ- isomers≤0.3 mg/kg	未檢出 Not detected<br>符合規定 Conform
- 林丹 Lindane	≤0.6 mg/kg	未檢出 Not detected<br>符合規定 Conform
- 五氯硝基苯 Quintozene	五氯硝基苯，五氯苯胺及甲基五氯硫基苯之和≤1.0 毫克/千克<br>Sum of quintozene, pentachloroaniline and methyl pentachlorophenyl sulphide≤1.0 mg/kg	未檢出 Not detected<br>符合規定 Conform
8. 微生物限度#<br>Microbial Limit		
- 細菌數 Microbial Count	≤500cfu/g	＜10cfu/g<br>符合規定 Conform
- 霉菌及酵母菌<br>Mould and Yeast Count	≤100cfu/g	＜10cfu/g<br>符合規定 Conform
- 大腸埃希菌 Escherichia Coli	不得檢出/克 Not detected/g	未檢出/克 Not detected/g<br>符合規定 Conform

****

編制 Report compiled by：[signature] 審核 Reviewed by：[signature]

註：Remarks

1、 報告無“檢驗/檢測報告專用章”和缺騎縫章無效。

Test report without the stamp of "For Test/Test Report Only" is not authorized.

2、 複製的報告未重新加蓋“檢驗/檢測報告專用章”及騎縫章無效。

Certified copy of test reportwithout the stamp of"For Test/Test Report Only" is not authorized.

3、 報告無編制、審核及本中心代表簽字無效。

Test report must be authorized by the signature of the bodies of preparation, verification and representative of the testing centre.

4、 報告塗改、缺頁無效。

Test report with modification or lacking of page(s) is not authorized.

5、 對報告若有異議，請於收到報告之日起十五日內向檢驗單位提出書面申訴，否則按認可檢驗報告處理。本中心異議受理電話 0771-3218026。

The client is aggrieved by the test result. A request for review shall state in writing the reasons relied upon and shall be made to the testing centre within 15 days after receipt of the authorized test report. No requirement will be accepted after the definite time. Inquiry hot line is 0771-3218026.

6、 送樣委託檢驗，樣品名稱為委託單位自報名稱，報告僅對來樣負責。部份複製檢驗/檢測報告無效。

Name of tested product is provided by the client. The report will refer only to the sample tested. Copy of partial of the test report is not authorized.

# - 其他微生物限度要求: Other requirements for Microbial Limit Test

1.霉菌及酵母菌: 固體製劑及不含糖、王漿、蜂蜜的液體製劑或半固體製劑檢查霉菌；含糖、王漿、蜂蜜的液體製劑或半固體製劑檢查霉菌和酵母菌。

A)Mould and Yeast Count: For dose in solid form and liquid form not containing sugar, royal jelly, honey or semi-solid form, mould count should be tested. For dose in liquid form containing sugar, royal jelly, honey or semi-solid form, both mould count and yeast count should be tested.

2.含有動物類原藥材的口服製劑，不得檢出沙門氏菌；含動物角、王漿、蜂蜜、阿膠的口服製劑, 則毋須進行有關檢定。

For oral preparations containing animal-origins raw ingredients, Salmonella should not be detected. For oral preparations containing animal horn, royal jelly, bee honey and CollaCoriiAsini, test for Salmonella may be exempted.

*** End of Report 報告完 ***
