# Supplementary material for: Inhibitory effect of a weight-loss Chinese herbal formula RCM-107 on pancreatic α-amylase activity: Enzymatic and in silico approaches
Source: PLoS One. 2020 Apr 29;15(4):e0231815. doi: 10.1371/journal.pone.0231815 (PMC7190128; doi:10.1371/journal.pone.0231815)
Supplement: S1 Quality assessments — (PDF) [file pone.0231815.s008.pdf]

Ferngrove Pharmaceuticals Australia

Finished Product Specification

Product name	**Slimming Plus**		
Sponsor/Customer Name	Tong Kang Lee Chinese Medical Center Pty Ltd	**Prepared by Technical Officer**	
AUST L	xxxxxx	Name: Yueting Lu	5/12/201
Product code - Ferngrove	HC-CS150-TK	**Checked by Technical Officer**	
Document No.	STP-02-2193-1	Name : Nagaraj Manda	
Version No.	2		
Issue date	05-Dec-16	**Approved by Regulatory Manager**	
Review date	05-Dec-19	Name : Eugene Ng	5/12/

1. General Specification

No.	Fields	Specification	Reference
1.1	Dosage form	Capsule, hard gel.	
1.2	Route of Administration	Oral	
1.3	Physical appearance	"00" clear hard gel capsule containing beige color mottled free flowing powder.	
1.4	Fill weight	680 mg±7.5%	TGO78
1.5	Total weight	802 mg±7.5%	In house
1.6	Uniformity of Mass	Complies to Current Therapeutic Goods Order	TGO78
1.7	Disintegration	Not more than 30 minutes.	BP.

2. Master Formulation

No.	Active Ingredient	RM Code	Label claim mg/capsule	Actual mg/capsule	Overage %	Release limit %	Reference standard
2.1	**Camellia sinensis** (Green Tea) leaf Ext. Conc. dry 10:1 in 100% Water Purified.	HB034	_	150	_	QBI.	Manuf.
	Equiv. Camellia sinensis leaf dry		*1500*	_			
	Contains Caffine			12			
2.2	**Senna tora** (Jue Ming Zi) fruit Ext. Vacuum Conc. Vacuum dry 10:1 in 100% Water Purified, Excipients : Nil	HB510	_	50	_	QBI.	Manuf.
	Equiv.Senna tora fruit dry		*500*	_			
2.3	**Styphnolobium japonicum** (Huai Hua Mi) flower Ext. Vacuum Conc., Vacuum dry 10:1 in 100% Water Purified.	HB512		50	_	QBI.	Manuf.
	Equiv. Styphnolobium japonicum flower dry		*500*				
2.4	**Plantago Asiatic** (Che Qian Zi) seed Ext. Vacuum Conc. dry 10:1 in 60% E:W	HB514		50	_	QBI.	Manuf.
	Equiv. Plantago Asiatic seed dry		*500*				
	Contains 1% of Silicon dioxide						
2.5	**Gardenia Jasminoides** (Shan Zhi Zi) seed Ext. Vacuum Conc. dry 10:1 in 75% E:W	HB513	_	50	_	QBI.	Manuf.
	Equiv. Gardenia Jasminodes seed dry		*500*	_			

No.	Active ingredient	RM Code	Quantity mg/capsule	Actual mg/capsule	Overage %	Release limit %	Reference standard
2.6	**Nelumbo nucifera** (Lotus) leaf Ext. dry Conc. 10:1 in 70% E:W	HB518	_	50	_	QBI.	Manuf.
	Equiv. Nelumbo nucifera leaf dry		500	_			
2.7	**Poria Cocos** (Fu Ling) Fungi mushroom Ext. dry Conc. 10:1 in 65% E:W	HB516	_	50	_	QBI.	Manuf.
	Equiv. Poria Cocos Fungi mushroom dry		500				
	Contains Maltodextrin as Excipient.			_			
2.8	**Alisma Orientale** (Ze Xie) rhizome Ext. dry Conc. 10:1 in 45% E:W.	HB517	_	50	_	QBI.	Manuf.
	Equiv. Alisma Orientale rhizome dry		500	_			
	Ingredient weight			500.00			

No.	Excipient	RM Code	Quantity mg/capsule	Actual mg/capsule	Overage %	Release limit %	Reference standard
2.9	Silica colloidal anhydrous	EX005	12	_	_	_	BP.
2.1	Calcium hydrogen phosphate dihydrate	EX008T	87	_	_	_	USP.
2.11	Magnesium stearate	EX004	11	_	_	_	BP.
2.12	Microcrystalline cellulose	EX019	70	_	_	_	BP.
	Excipients weight		180.00				

No.	Capsule shell	RM Code	Quantity mg/capsule	Actual per capsule	Overage %	Release limit %	Reference standard
2.13	Hard gel capsule clear size "00"	CP003/ CP008	122	122	_	_	Manuf.
	Capsule weight			122			

3. Fill Weight & Total Weight

No.	Item	Weight mg/capsule	Release limit (mg)	Reference standard
3.1	Active ingredients	500.00	_	_
3.2	Excipients	180.00	_	_
	**Fill Weight**	680.00	629 _ 731	TGO78
3.3	Capsule weight	122.00	_	_
	**Total weight**	802.00	742 _ 862	In house

4. TSE Assessment (Transmissible Spongiform Encephalopathy)

No.	Item	Specific Animal Ingredient	Statement
4.1	TSE assessment required	Gelatin	TSE free
4.2	TGA preclearance number	_	_

Ferngrove Pharmaceuticals Australia

5. Microbial Release Limits

No.	Item	Release limit	Reference standard
5.1	Total Viable Count (TVC)	NMT. 1×10^4cfu/g	TGO77
5.2	Yeast and Mould Count	NMT. 1×10^2cfu/g	TGO77
5.3	Bile-tolerant Gram negative bacteria	NMT. 1×10^2cfu/g	TGO77
5.4	Salmonella	Not detected/10g	TGO77
5.5	Escherichia coli	Not detected/g	TGO77
5.6	Staphylococcus aureus	Not detected/g	TGO77

The first 3 batches will be microbiologically tested for finished products to meet TGO77 and then as part of a rotational testing program 1 batch in every 10th batch manufactured will be tested thereafter.

6. Product Stability Information

No.	Item	Specification
6.1	Shelf life	Sponsor
6.2	Storage temperature	Store below 30°C
6.3	Storage condition	Store away from direct heat & sunlight

7. GMO Status & Free From Statement

7.1 GMO status - free.

7.2 Contains animal sourced ingredient - gelatin from bovine hide & sourced from China.

8. Revision History

No.	Amendment details	Section & Item	Revision date
8.1	New document	All	04-Nov-16
8.2	Version 2	Remove HB511, add HB518	05-Dec-16

9. Sponsor/Customer Approval:

I hereby certify that the finished product specification complies with market authorisation and stability data exists to support the shelf life based on scientific ground.

Product/Brand Name:	Slimming Plus
AUST L No:	xxxxxx
Sponsor/Customer Name:	Tong Kang Lee Chinese Medical Center Pty Ltd
Expiry date:	(3) years from date of manufacture
Approved By:	KANG XIAO LI
Position:	DIRECTOR
Signature& date:	Cokangxiao 05. 12. 2016

"BP" = British Pharmacopoeia; "BHP" = British Herbal Pharmacopoeia; "USP" = United States Pharmacopoeia; "EP" = European Pharmacopoeia; "BPC" = British Pharmacopoeia Codex; "Manuf." Manufactuerer's specification; "TGA" = Therapeutic Goods Administration; "MI" = Merck Index; "FCC" = Food Chemicals Codex; "CI" = Colour Index. "QBI" = Quantified by Input, Refer to the Australian Department of Health and Ageing Therapeutic Goods Administration's document "Guidance on the Use of the term 'Quantified by Input' for Complementary Medicines". Please note that all pharmacopeia referenced are current edition, unless otherwise stated.

Release limit test may be conducted by contract laboratories:

Ferngrove Pharma Aust P/L 5 Ferngrove Place, South Granville, NSW2142- Chemical & Physical testing (MI-19092007-LI-002109-11)

Naturalab Pty Ltd Level 2/111 Stephen Road,Botany NSW 2019 - Chemical & Physical testing (TGA No. MI-20062007-LI-001909-11)

AMS (Eurofins AMS) Laboratories Pty Ltd 8 Rachael Close , Silverwater NSW 2128 - Microbial testing (TGA No : MI-15112007-LI-002191-11)

Southern Cross Plant Science T Block Level 3, Military Road, Lismore, NSW,2480 - Chemical Testing (TGA No:181048)

Merieux NutriSciences (Silliker) 391 Park Road, Regents Park,NSW 2143 - Microbial testing (TGA No. MI-04072005-LI-00664-2)

End of documentation
