# Supplementary material for: Inhibitory effect of a weight-loss Chinese herbal formula RCM-107 on pancreatic α-amylase activity: Enzymatic and in silico approaches
Source: PLoS One. 2020 Apr 29;15(4):e0231815. doi: 10.1371/journal.pone.0231815 (PMC7190128; doi:10.1371/journal.pone.0231815)
Supplement: S1 Protocol — (DOCX) [file pone.0231815.s009.docx]

**Pancreatic alpha amylase assay protocol.**

**1) Prepare sample stock and serial dilution (get Distilled water (500mL) and DMSO (1-2 mL) in a beaker, vortex buffer)** 20mg (0.02g) in 4mL (2% DMSO: 80µL DMSO + 3000+920 µL water) filter (1x15mL falcon tube+7 **small centrifuge tubes**) and vortex/sonicate 10min until fully dissolved. 5mg/mL, 2 %DMSO **25 µL**

| RCM-107+ formula | Tube 0(stock) | Tube 1 | Tube 2 | Tube3 | Tube 4 | Tube 5 | Tube6 | Tube 7 |
| --- | --- | --- | --- | --- | --- | --- | --- | --- |
| Volume taken from previous tube(µL) | Stock | 640 | 625 | 600 | 333 | 500 | 600 | 333 |
| Volume of buffer added(µL) | Stock | 360 | 375 | 400 | 667 | 500 | 400 | 667 |
| Concentration of SP+ formula /tube(µg/mL) | 5,000 | 3200 | 2000 | 1200 | 400 | 200 | 120 | 40 |
| Final concentration in the well(µg/mL) | Stock | 800 | 500 | 300 | 100 | 50 | 30 | 10 |
| Final volume of each tube(mL) | 4 | 1 | 1 | 1 | 1 | 1 | 1 | 1 |

Plate 2. Individual Stock 5mg/mL 20mg (0.02g) in 4mL (2%: 80µL DMSO + 3000+920 µL water**)** (1x15mL falcon tubes+ 1 small centrifuge tubes for each herbs) 5mg/mL, 4%DMSO

| Individual herbs | Tube 0(stock) | Tube 1 |
| --- | --- | --- |
| Volume taken from previous tube(µL) | Stock | 240 |
| Volume of buffer added(µL) | Stock | 760 |
| Concentration of SP+ formula /tube(µg/mL) | 5,000 | 1200 |
| Final concentration in the well(µg/mL) | Stock | 300 |
| Final volume of each tube(mL) | 4 | 1 |

**2) Prepare Acarbose stock and dilution 25**µL

1. Weight 20mg (0.02g) Acarbose in 4mL (2%: 80µL DMSO + 3000+920 µL buffer) (1x15mL falcon tube+1 **small centrifuge tubes**)

| Individual herbs | Tube 0(stock) | Tube 1 |
| --- | --- | --- |
| Volume taken from previous tube(µL) | Stock | 240 |
| Volume of buffer added(µL) | Stock | 760 |
| Concentration of SP+ formula /tube(µg/mL) | 5,000 | 1200 |
| Final concentration in the well(µg/mL) | Stock | 300 |
| Final volume of each tube(mL) | 4 | 1 |

**3) Prepare substrate stock and dilution (in dark)—prepare fresh for each plate 25 µL 1 x15mL falcon tube**

1. Take **100µL** of **Component C** (50mM sodium acetate buffer/**solvent**, pH4.0 ) and add in 1 vial from **Component A** (1 mg substrate)
2. Vortex for 20 seconds each time for 3 min (leave in room temperature) until it’s dissolved.
3. Add **900µL** of 100mM **sodium phosphate buffer** to the vial of **component A**.
4. Mix well by vortex for 20s, in dart and place in room temperature.
5. Don’t freeze the substrate, can be stored at 4 ͦC for a few days but not recommended.
6. Place **4mL** of buffer in 15mL falcon tube (**Tube 1**) and take **1mL** from **Vial 1**-------- Final working solution **200µg/mL**

**Vial 1**

**Tube 1**

5 mL

1mL

Take **1mL** out from **Vial 1** and add **4mL** 100mM sodium phosphate buffer

**(1mg/mL) (200µg/mL)**

**Use pipette to get 2.5mL (2000 µL +500 µL out when putting them into the plate)**

Substrate working solution 200µg/mL, total volume 5mL

C1V1=C2V2

V1= 0.2mg/mL x5mL

1mg/mL

=1mL

**4) Prepare Amylase working solution (48mU/mL) ---Gentle vortex and inversion**

1. Dilute 10^7^mU/mL amylase (50mg/5mL; 1000U/mg) to 10^3^mU/mL amylase stock solution. (Need 3x15mL falcon tubes for 3 separate dilution, 6 small tubes)

Take **100µL** from tube0;

Add **9.9mL** **(9000µL+900µL)** 100mM buffer

Take **100µL** from previous vial;

Add **9.9mL** **(9000 µL+900 µL)** 100mM buffer

*10mL*

10 mL

10^7^mU/mL Tube0: 10^5^mU/mL Tube 00: 10^3^mU/mL

1^st^ dilution: C1v1=C2V2

V1=C2V2/C1

= 10^5^mU/mL x 10mL/ 10^7^mU/mL

= 0.1mL=100µL

10mL

Tube00

2^nd^ dilution: C1v1=C2V2

Take **480µL** from Tube00;

Add **9.52mL (9000µL+520 µL)** buffer

V1=C2V2/C1

= 10^3^mU/mL x 10mL/ 10^5^mU/mL Tube 00: 10^3^mU/mL Tube 1: 48mU/mL

= 0.1mL=100µL

3^rd^= 16mU/mLx5mL/10^3^mU/mL =0.08mL=80µL

2. Make **working solution** for amylase (25 µL, 48 mU/mL/tube; 12mU/mL/Well)

3. Prepare standard curve for amylase (See table)--25µL each in the well use from tube 3-tube7 as standard curve

|  | Tube00 | Tube3 | Tube4 | **Tube 5(W)** | Tube6 | Tube7 |
| --- | --- | --- | --- | --- | --- | --- |
| Volume taken out from previous tube(µL) | Stock | 80 | 800 | **750** | 667 | 500 |
| Buffer need to be added(µL) | Stock | 920 | 200 | **250** | 333 | 500 |
| Concentration of Amylase /tube(mU/mL) | 1000 | 80 | 64 | **48** | 32 | 16 |
| Final concentration of amylase /well (mU/mL) | Stock | 20 | 16 | **12** | 8 | 4 |
| Volume per tube(mL) | 10 | 1 | 1 | **1** | 1 | 1 |

**10 mM PBS in 500mL pH 6.9(6.94) 1 tablet in 500mL distilled water**

| **Ingredients** | MW | Concentration |
| --- | --- | --- |
| **Sodium phosphate** | 95 | 10mM |
| **Potassium Chloride (KCl)** | 75 | 0.00268M |
| **Sodium Chloride (NaCl)** | 58 | 0.14M |

+1mM calcl2: 27.5mg

pH 6.9

| **PLATE(1)** | **1** | **2** | **3** | **4** | **5** | **6** | **7** | **8** | **9** | **10** | **11** | **12** |
| --- | --- | --- | --- | --- | --- | --- | --- | --- | --- | --- | --- | --- |
| **A** |  | **Blank**  Nothing in the well | SPS (0)  B 50 µL(1.6)  SS 25 µL  ES 25 µL  (Neg Control) | SPS (0)  B 50 µL(1.6)  SS 25µL  ES 25 µL  (Neg Control) | SPS (0)  B 50 µL(1.6)  SS 25 µL  ES 25 µL  (Neg Control) | **Blank**  SS 25 µL  B 75 µL(1.6) | **Blank**  SS 25 µL  B 75 µL(1.6) | **Blank**  B 100 µL  (1.6) | **Blank**  B 100 µL  (1.6) | **Blank**  B 75 µL  (1.6)  ES 25 µL W | GFS (40)  25 µL  B 75 µL | GFS (120)  25 µL  B 75 µL |
| **B** | SPS (40)  25µL  B 75 µL | SPS (40)  25µL  B 75 µL | SPS (40)  25µL  ES 25 µL  SS 25 µL  B 25 µL | SPS (40)  25µL  ES 25 µL  SS 25 µL  B 25 µL | SPS (40)  25µL  ES 25 µL  SS 25 µL  B 25 µL | GFS (40)  25 µL  ES 25 µL  SS 25 µL  B 25 µL | GFS (40)  25 µL  ES 25 µL  SS 25 µL  B 25 µL | GFS (40)  25 µL  ES 25 µL  SS 25 µL  B 25 µL | ***ES(16)***  25 µL  B 50 µL(1.6)  ***SS 25*** µL | ***ES(16)***  25 µL  B 50 µL(1.6)  ***SS 25*** µL | ***ES(16)***  25 µL  B 75 µL(1.6) | GFS (200)  25 µL  B 75 µL |
| **C** | SPS (120)  25 µL  B 75 µL | SPS (120)  25 µL  B 75 µL | SPS (120)  25 µL  ES 25 µL  SS 25 µL  B 25 µL | SPS (120)  25 µL  ES 25 µL  SS 25 µL  B 25 µL | SPS (120)  25 µL  ES 25 µL  SS 25 µL  B 25 µL | GFS (120)  25 µL  ES 25 µL  SS 25 µL  B 25 µL | GFS (120)  25 µL  ES 25 µL  SS 25 µL  B 25 µL | GFS (120)  25 µL  ES 25 µL  SS 25 µL  B 25 µL | ***ES(32)***  25 µL  B 50 µL(1.6)  ***SS 25*** µL | ***ES(32)***  25 µL  B 50 µL(1.6)  ***SS 25*** µL | ***ES(32)***  25 µL  B 75 µL(1.6) | GFS (400)  25 µL  B 75 µL |
| **D** | SPS (200)  25 µL  B 75 µL | SPS (200)  25 µL  B 75 µL | SPS (200)  25 µL  ES 25 µL  SS 25 µL  B 25 µL | SPS (200)  25 µL  ES 25 µL  SS 25 µL  B 25 µL | SPS (200)  25 µL  ES 25 µL  SS 25 µL  B 25 µL | GFS (200)  25 µL  ES 25 µL  SS 25 µL  B 25 µL | GFS (200)  25 µL  ES 25 µL  SS 25 µL  B 25 µL | GFS (200)  25 µL  ES 25 µL  SS 25 µL  B 25 µL | ***ES(48)***  25 µL  B 50 µL(1.6)  ***SS 25*** µL | ***ES(48)***  25 µL  B 50 µL(1.6)  ***SS 25*** µL | ***ES(48)W***  25 µL  B 75 µL(1.6) | GFS (1200)  25 µL  B 75 µL |
| **E** | SPS (400)  25 µL  B 75 µL | SPS (400)  25 µL  B 75 µL | SPS (400)  25 µL  ES 25 µL  SS 25 µL  B 25 µL | SPS (400)  25 µL  ES 25 µL  SS 25 µL  B 25 µL | SPS (400)  25 µL  ES 25 µL  SS 25 µL  B 25 µL | GFS (400)  25 µL  ES 25 µL  SS 25 µL  B 25 µL | GFS (400)  25 µL  ES 25 µL  SS 25 µL  B 25 µL | GFS (400)  25 µL  ES 25 µL  SS 25 µL  B 25 µL | ***ES(64)***  25 µL  B 50 µL(1.6)  ***SS 25*** µL | ***ES(64)***  25 µL  B 50 µL(1.6)  ***SS 25*** µL | ***ES(64)***  25 µL  B 75 µL(1.6) | GFS (2000)  25 µL  B 75 µL |
| **F** | SPS (1200)  25 µL  B 75 µL | SPS (1200)  25 µL  B 75 µL | SPS (1200)  25 µL  ES 25 µL  SS 25 µL  B 25 µL | SPS (1200)  25 µL  ES 25 µL  SS 25 µL  B 25 µL | SPS (1200)  25 µL  ES 25 µL  SS 25 µL  B 25 µL | GFS (1200)  25 µL  ES 25 µL  SS 25 µL  B 25 µL | GFS (1200)  25 µL  ES 25 µL  SS 25 µL  B 25 µL | GFS (1200)  25 µL  ES 25 µL  SS 25 µL  B 25 µL | ***ES(80)***  25 µL  B 50 µL(1.6)  ***SS 25*** µL | ***ES(80)***  25 µL  B 50 µL(1.6)  ***SS 25*** µL | ***ES(80)***  25 µL  B 75 µL(1.6) | GFS (3200)  25 µL  B 75 µL |
| **G** | SPS (2000)  25 µL  B 75 µL | SPS (2000)  25 µL  B 75 µL | SPS (2000)  25 µL  ES 25 µL  SS 25 µL  B 25 µL | SPS (2000)  25 µL  ES 25 µL  SS 25 µL  B 25 µL | SPS (2000)  25 µL  ES 25 µL  SS 25 µL  B 25 µL | GFS (2000)  25 µL  ES 25 µL  SS 25 µL  B 25 µL | GFS (2000)  25 µL  ES 25 µL  SS 25 µL  B 25 µL | GFS (2000)  25 µL  ES 25 µL  SS 25 µL  B 25 µL | AS(1200)  25 µL  ES 25 µL  SS 25 µL  B 25 µL | AS(1200)  25 µL  ES 25 µL  SS 25 µL  B 25 µL | AS (1200)  25 µL  ES 25 µL  SS 25 µL  B 25 µL | AS(1200)  25 µL  B 75 µL |
| **H** | SPS (3200)  25 µL  B 75 µL | SPS (3200)  25 µL  B 75 µL | SPS (3200)  25 µL  ES25 µL  SS50 µL  B 25 µL | SPS (3200)  25 µL  ES25 µL  SS50 µL  B 25 µL | SPS (3200)  25 µL  ES25 µL  SS50 µL  B 25 µL | GFS (3200)  25 µL  ES 25 µL  SS 25 µL  B 25 µL | GFS (3200)  25 µL  ES 25 µL  SS 25 µL  B 25 µL | GFS (3200)  25 µL  ES 25 µL  SS 25 µL  B 25 µL |  |  |  |  |

| **PLATE(2)** | **1** | **2** | **3** | **4** | **5** | **6** | **7** | **8** | **9** | **10** | **11** | **12** |
| --- | --- | --- | --- | --- | --- | --- | --- | --- | --- | --- | --- | --- |
| **A** | SPS (400)  25 µL  B 75 µL | SPS (400)  25 µL  B 75 µL | SPS (0)  B 50µL(0.16)  SS 25 µL  ES 25 µL  (Neg Control) | SPS (0)  B 50µL(0.16)  SS 25µL  ES 25 µL  (Neg Control) | SPS (0)  B 50µL(0.16)  SS 25 µL  ES 25 µL  (Neg Control) | **Blank**  SS 25 µL  B 75 µL  (0.16) | **Blank**  SS 25 µL  B 75 µL  (0.16) | **Blank**  B 100 µL  (0.16) | **Blank**  B 100 µL  (0.16) | **Blank**  B 75 µL(0.16)  ES 25 µL | **Blank**  B 75 µL0.16  ES 25 µL |  |
| **B** | AS (400)  25µL  B 75 µL | AS(400)  25µL  B 75 µL | SPS (400)  25 µL  ES 25 µL  SS 25 µL  B 25 µL | SPS (400)  25 µL  ES 25 µL  SS 25 µL  B 25 µL | SPS (400)  25 µL  ES 25 µL  SS 25 µL  B 25 µL | AS(400)  25 µL  ES 25 µL  SS 25 µL  B 25 µL | AS(400)  25 µL  ES 25 µL  SS 25 µL  B 25 µL | AS(400)  25 µL  ES 25 µL  SS 25 µL  B 25 µL | PC(400)  25 µL  ES 25 µL  SS 25 µL  B 25 µL | PC(400)  25 µL  ES 25 µL  SS 25 µL  B 25 µL | PC(400)  25 µL  ES 25 µL  SS 25 µL  B 25 µL |  |
| **C** | PC(400)  25 µL  B 75 µL | PC(400)  25 µL  B 75 µL | LFS (400)  25 µL  ES 25 µL  SS 25 µL  B 25 µL | LFS (400)  25 µL  ES 25 µL  SS 25 µL  B 25 µL | LFS (400)  25 µL  ES 25 µL  SS 25 µL  B 25 µL | PASS (400)  25 µL  ES 25 µL  SS 25 µL  B 25 µL | PASS (400)  25 µL  ES 25 µL  SS 25 µL  B 25 µL | PASS (400)  25 µL  ES 25 µL  SS 25 µL  B 25 µL | ARS (400)  25 µL  ES 25 µL  SS 25 µL  B 25 µL | ARS (400)  25 µL  ES 25 µL  SS 25 µL  B 25 µL | ARS (400)  25 µL  ES 25 µL  SS 25 µL  B 25 µL |  |
| **D** | LFS(400)  25 µL  B 75 µL | LFS(400)  25 µL  B 75 µL | CSS (400)  25 µL  ES 25 µL  SS 25 µL  B 25 µL | CSS (400)  25 µL  ES 25 µL  SS 25 µL  B 25 µL | CSS (400)  25 µL  ES 25 µL  SS 25 µL  B 25 µL | SFS (400)  25 µL  ES 25 µL  SS 25 µL  B 25 µL | SFS (400)  25 µL  ES 25 µL  SS 25 µL  B 25 µL | SFS (400)  25 µL  ES 25 µL  SS 25 µL  B 25 µL | GFS (400)  25 µL  ES 25 µL  SS 25 µL  B 25 µL | GFS(400)  25 µL  ES 25 µL  SS 25 µL  B 25 µL | GFS (400)  25 µL  ES 25 µL  SS 25 µL  B 25 µL |  |
| **E** | PASS(400)  25 µL  B 75 µL | PASS(400)  25 µL  B 75 µL | GTS (400)  25 µL  ES 25 µL  SS 25 µL  B 25 µL | GTS (400)  25 µL  ES 25 µL  SS 25 µL  B 25 µL | GTS (400)  25 µL  ES 25 µL  SS 25 µL  B 25 µL |  | ***ES(16)***  25 µL  B 50 µL 0.16  ***SS 25*** µL | ***ES(32)***  25 µL  B 50 µL0.16  ***SS 25*** µL | ***ES(48)***  25 µL  B 50 µL0.16  ***SS 25*** µL | ***ES(64)***  25 µL  B 50 µL0.16  ***SS 25*** µL | ***ES(80)***  25 µL  B 50 µL0.16  ***SS 25*** µL |  |
| **F** | ARS(400)  25 µL  B 75 µL | ARS(400)  25 µL  B 75 µL | SPS (0)  B 50µL  SS 25 µL  ES 25 µL | SPS (0)  B 50µL  SS 25µL  ES 25 µL | SPS (0)  B 50µL  SS 25 µL  ES 25 µL |  | ***ES(16)***  25 µL  B 50 µL0.16  ***SS 25*** µL | ***ES(32)***  25 µL  B 50 µL0.16  ***SS 25*** µL | ***ES(48)***  25 µL  B 50 µL0.16  ***SS 25*** µL | ***ES(64)***  25 µL  B 50 µL0.16  ***SS 25*** µL | ***ES(80)***  25 µL  B 50 µL0.16  ***SS 25*** µL |  |

B buffer ES: enzyme

SS substrate

Twenty-five microliters of each individual herbal extract at the final concentration of 300 µg/mL, the RCM-107 formula extraction (10-800µg/mL) and 25 µL of the PPA solution (final concentration 12mU/mL) were pre-incubated in a 96-well plate with 25 µL PBS buffer (The incubation buffer consisted of 10mM sodium phosphates, 2.68mM KCl, 140 mM NaCl and 1mM CaCl_2_, pH 6.9), in dark at room temperature (25 ºC) for 20 min.

To initiate the enzyme reaction, 25µL of 200 µg/mL DQ^TM^ starch solution was added.

Fluorescence was measured with the CLARIOstar^®^ microplate reader (BMG labtech) at an excitation and emission wavelength of 485nm and 530nm, respectively over 30 min
